# Limited simultaneous nitrification-denitrification (SND) in aerobic granular sludge systems treating municipal wastewater: Mechanisms and practical implications

**DOI:** 10.1016/j.wroa.2020.100048

**Published:** 2020-02-27

**Authors:** Manuel Layer, Mercedes Garcia Villodres, Antonio Hernandez, Eva Reynaert, Eberhard Morgenroth, Nicolas Derlon

**Affiliations:** a Eawag: Swiss Federal Institute of Aquatic Science and Technology, Überlandstrasse 133, CH-8600, Dübendorf, Switzerland; b Institute of Environmental Engineering, ETH Zürich, CH-8093, Zürich, Switzerland

**Keywords:** Aerobic granular sludge, Municipal wastewater, Simultaneous nitrification denitrification, Total nitrogen removal, Aeration strategy

## Abstract

Simultaneous nitrification-denitrification (SND) is, in theory, a key advantage of aerobic granular sludge systems over conventional activated sludge systems. But practical experience and literature suggests that SND and thus total nitrogen removal are limited during treatment of municipal wastewater using AGS systems. This study thus aims at quantifying the extent and understanding the mechanisms of SND during treatment of municipal wastewater with aerobic granular sludge (AGS) systems. Experiments (long-term and batch-tests) as well as mathematical modelling were performed. Our experimental results demonstrate that SND is significantly limited during treatment of low-strength municipal wastewater with AGS systems (14–39%), while almost full SND is observed when treating synthetic influent containing only diffusible substrate (90%). Our simulations demonstrate that the main mechanisms behind limited SND are (1) the dynamics of anoxic zone formation inside the granule, (2) the diffusibility and availability of electron-donors in those zones and (3) the aeration mode. The development of anoxic zones is driven by the utilisation of oxygen in the upper layers of the granule leading to transport limitations of oxygen inside the granule; this effect is closely linked to granule size and wastewater composition. Development of anoxic zones during the aerobic phase is limited for small granules at constant aeration at bulk dissolved oxygen (DO) concentration of 2 mgO_2_ L^−1^, and anoxic zones only develop during a brief period of the aerated phase for large granules. Modelling results further indicate that a large fraction of electron-donors are actually utilised in aerobic rather than anoxic redox zones – in the bulk or at the granule surface. Thus, full SND cannot be achieved with AGS treating low strength municipal wastewater if a constant DO is maintained during the aeration phase. Optimised aeration strategies are therefore required. 2-step and alternating aeration are tested successfully using mathematical modelling and increase TN removal to 40–79%, without compromising nitrification, and by shifting electron-donor utilisation towards anoxic redox conditions.

## Highlights

• We studied SND in AGS systems.
•Limited SND was observed in municipal wastewater fed AGS systems.
•Availability and dynamics of anoxic zones and electron-donors limit SND in AGS.
•Larger granules provide larger anoxic zones and hence improved SND performance.
•Optimised aeration increases denitrification performance of AGS significantly.

## Graphical abstract

**Figure:**
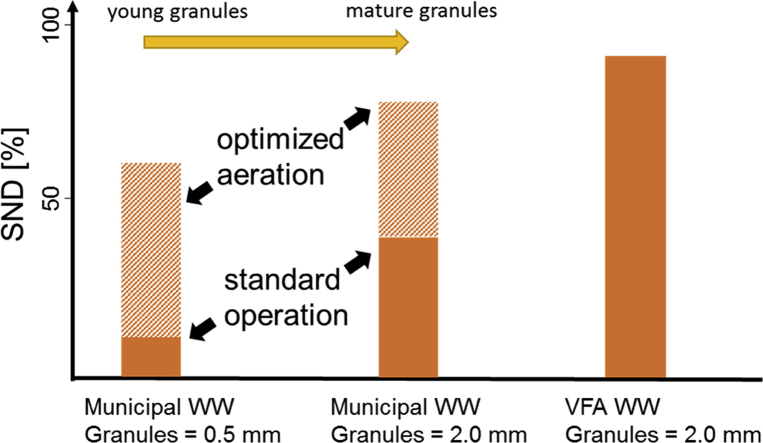

## 1 Introduction

van Loosdrecht and Brdjanovic, 2014 In AGS systems, mass transfer is limited by diffusion, which leads to concentration gradients of electron-donors/final acceptors within the granules. During the aerated phase, an oxygen gradient develops within the granules whereby the outer layers are aerobic and the inner core is anoxic or anaerobic (De Kreuk et al., 2007a). Those different redox conditions within the granules allow nitrifying, denitrifying and facultative anaerobic organisms to coexist (Winkler et al., 2013). As a result, it is usually well accepted that simultaneous nitrification and denitrification (SND) is a key feature of AGS and that SND is the main nitrogen removal pathway in AGS systems (De Kreuk et al., 2005; Adav et al., 2008; Pronk et al., 2015).

**Fig. 1:**
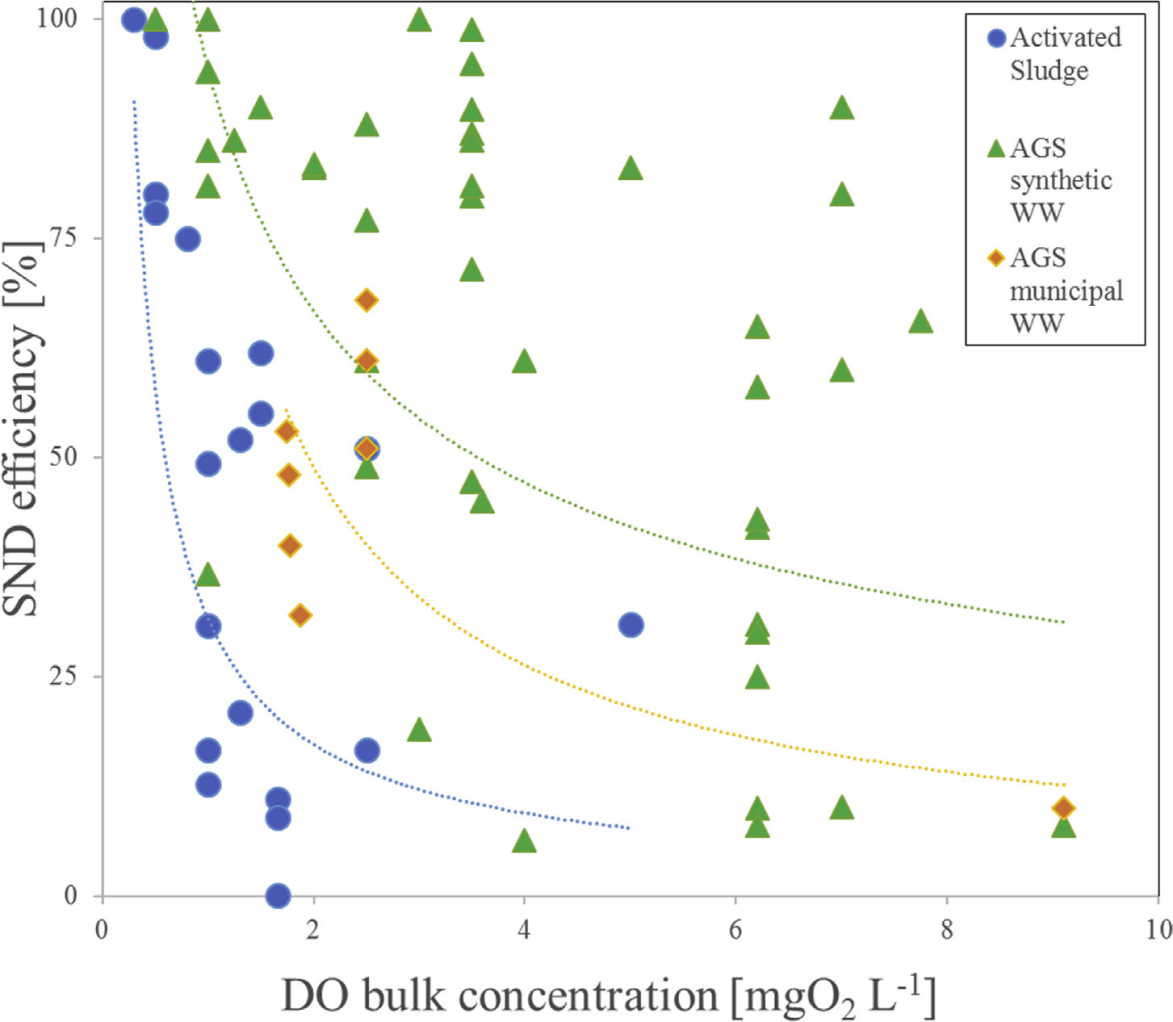
SND efficiencies for different DO bulk concentrations reported in literature for activated sludge systems and aerobic granular sludge fed with synthetic acetate/propionate based influent (AGS synthetic) or MWW (AGS municipal WW) (Mosquera-Corral et al., 2005; Kishida et al., 2006; Lochmatter et al., 2013; Kocaturk and Erguder, 2016; Isanta et al., 2012; He et al., 2018; He et al., 2019; He et al., 2017; Wang et al., 2015; Semerci and Hasılcı, 2016; Rollemberg et al., 2019; Pronk et al., 2015; Wang et al., 2009; De Kreuk and Van Loosdrecht, 2006; Liu et al., 2011; Świątczak and Cydzik-Kwiatkowska, 2018; Derlon et al., 2016; Lashkarizadeh et al., 2015; Liu et al., 2010; Ni et al., 2009; Wagner et al., 2015; Pochana and Keller, 1999; Third et al., 2003; Zeng et al., 2003; Wang et al., 2015; Lo et al., 2010; Marin et al., 2019). Data on SND were collected directly from literature (whenever given), or calculated from batch-test data or in-cycle concentration profiles of N-species (Supplementary Information S1).

In theory, complete total nitrogen (TN) removal via SND could be achieved in one single reactor and within a single aerobic phase of the SBR cycle. However, when analysing data from literature, it is less evident that high TN removal via SND occurs in AGS systems treating municipal wastewater (MWW) (Fig. 1). SND efficiencies reported for lab-scale AGS systems fed with synthetic influent (mostly volatile fatty acids, VFA) are highly variable. Values ranging from 10 to 100% are reported for different dissolved oxygen (DO) concentrations in the bulk liquid (Kocaturk and Erguder, 2016; De Kreuk et al., 2005; Lochmatter et al., 2013). But for those systems, high SND efficiencies of more than 75% are typically observed for DO values below 4 mgO_2_ L^−1^ (Fig. 1). In AGS systems fed with MWW, SND efficiencies smaller than 50% on average are on the contrary reported (Fig. 1) (Pronk et al., 2015; De Kreuk and Van Loosdrecht, 2006; Świątczak and Cydzik-Kwiatkowska, 2018; Lashkarizadeh et al., 2015; Liu et al., 2010; Ni et al., 2009; Wagner et al., 2015). High variability of SND and/or low efficiencies lead to high TN and NO_3_^-^ effluent concentrations in the case of AGS-SBR operation without explicit anoxic phases. Low SND and TN removal of AGS is problematic in areas with stringent treatment requirements. It is therefore crucial to understand the extent and mechanisms of SND in order to further optimise TN removal of AGS systems.

SND requires (1) simultaneous occurrence of aerobic (for nitrification) and anoxic (for denitrification) redox conditions, and (2) electron-donor availability in anoxic redox conditions (for denitrification). Fig. 1 illustrates both effects. Lower DO concentrations generally tend to increase SND performances in both activated sludge and AGS systems (Pochana and Keller, 1999; Zeng et al., 2003; Third et al., 2003; He et al., 2017, 2019). In activated sludge flocs, anoxic micro-zones form due to high oxygen utilisation rates at the surface of the flocs (Li and Bishop, 2004), or by maintaining a bulk dissolved oxygen (DO) concentrations below the oxygen half-saturation constant (K_O2_) of denitrifying organisms (Daigger et al., 2007). Operation of activated sludge system at a DO set-point below K_O2_ thus results in SND without strict anoxic conditions. In comparison to activated sludge, larger SND efficiencies are observed for AGS systems across all DO concentrations (Fig. 1, green and orange dots). Higher SND in AGS systems results from the formation of anoxic zones inside the granules, which is driven by the limited diffusion of oxygen and simultaneous diffusion/production of NO_3_^-^. Another important observation is the distinct SND efficiency of AGS fed by synthetic WW – mostly composed of readily available VFA - vs. real MWW (Fig. 1). Indeed, the type of electron-donor and its availability partially determines SND efficiency (Pochana and Keller, 1999). Therefore, the distinct effects of the electron-donor availability, type and anoxic zone formation on SND in AGS systems need to be clarified.

AGS systems treating low strength MWW are typically characterized by slower start-up, more heterogeneous granule sizes, lower granule fractions, and an increased floc fraction in comparison to VFA-only WW fed AGS (Layer et al., 2019). The size of granules vary from d = 0.5 mm (or smaller) after few months (Ni et al., 2009; Liu et al., 2010; Wagner et al., 2015; Layer et al., 2019) up to d > 2 mm after few years of operation (Pronk et al., 2015). The granule size, together with the penetration depth of O_2_ in theory determines the extent of anoxic zone formation inside the deeper layers of the granule (Li et al., 2008). If the granule diameter impacts the penetration of O_2_, it is then key to evaluate the mechanisms of SND for both small (several hundred μm) and large (several mm) granules, representative of young and mature granules, respectively. The extent of anoxic zones in smaller granules might thus limit SND compared to larger granules. Another determinant of the extent of SND is the availability of (diffusible) electron-donors in the anoxic zones. Municipal WW contains a large fraction of electron-donors in the non-diffusible particulate form (X_B_), typically representing 50% of the total chemical oxygen demand (COD) (Metcalf and Eddy, 2014). If most of the electron-donors contained in MWW are not diffusible, it is then hypothesized that denitrification might also be limited during treatment of MWW. Understanding the distinct effects of the WW compositions and granule sizes on anoxic zone formation and electron-donor availability, and in turn on the SND and TN removal in AGS systems is therefore crucial.

The objectives of this study were therefore (1) to experimentally assess that SND and thus TN-removal is limited during treatment of low-strength MWW in comparison to 100%-VFA synthetic WW and (2) to then identify which mechanisms limit the extent of SND, *e.g.*, the dynamic of anoxic zone formation, the availability of different electron-donors inside the granules, and (3) to identify how to improve SND and TN removal by optimising the aeration strategies in AGS systems (2-step aeration, alternating aeration). Both experiments and mathematical modelling were conducted. SND and TN removal were quantified during long-term and batch experiments for different AGS systems fed with different WW to better understand the influence of DO, influent WW and sludge composition on the SND efficiency. An AGS model was then used to identify the effect of (1) electron-donor availability and contribution to SND and (2) anoxic zone formation inside the granules. The AGS model was then used to evaluate different aeration strategies in order to maximize TN removal.

## 2 Materials and methods

### 2.1 Experimental approach and reactor configuration

AGS were cultivated in 13 L column SBRs fed with 100%-VFA synthetic (R1), complex synthetic (R2), primary effluent (R3) and raw WW (R4), respectively (Layer et al., 2019). Influent composition in terms of electron-donor was either very simple (only soluble and highly diffusible organic acids) (R1), or increasingly complex in terms of electron-donor composition (R2, R3 and R4) (Table 1). All systems were operated at constant volume and SBR cycles were as follows: anaerobic plug-flow feeding (1.5 h), aerobic phase (4 h), settling (variable time), and excess sludge removal after settling. The total cycle length was 5.6 h. The DO concentration during the aerobic phase was controlled at a set-point of 2 mg O_2_ L^−1^ (constant DO). The settling and biomass properties of the different AGS grown with different influent compositions were characterised (Table 1). SND was assessed based on both long-term performances at constant DO concentrations and ex-situ batch tests at various DO concentrations. Long-term nitrification and denitrification performances and effluent quality (TN, NH_4_^+^, etc.) were monitored for 300–400 days. Batch-tests were performed on days 173 and 174 (R1), 195 and 196 (R2), 174 and 183 (R3), and 85 and 86 (R4) of operation, after establishment of granulation. For those batch tests, AGS was fed anaerobically during a regular SBR cycle, followed by aeration at different DO concentrations in fully mixed conditions (0.5–6.0 mgO_2_ L^−1^). Mixing was provided by a stirrer and aeration.

**Table 1:** Detailed influent composition, settleability (based on sludge volume index) and granule content of the four SBRs fed by 100%-VFA synthetic WW, complex synthetic WW, primary effluent WW and raw WW (Layer et al., 2019).

Reactor	100%-VFA synthetic WW	complex synthetic WW	primary effluent WW	raw WW
	R1	R2	R3	R4
Influent composition^a^	Synthetic influent (50% acetate, 50% propionate)	Synthetic influent (33% VFA, 33% fermentable substrate, 33% particulate substrate)	Low-strength municipal WW after primary sedimentation	Raw low-strength municipal WW
Total COD [mgCOD L^−1^]	582	503	331	469
Soluble COD [mgCOD L^−1^]	582	457	188	247
Soluble COD/particulate COD-ratio [−]	–	9.9	1.3	1.1
TN [mgN L^−1^]	43	44	33	41
NH_4_–N [mgN L^−1^]	40	20	24	29
Total COD/TN-ratio [mgCOD mgN^−1^]	13.5	11.4	10.0	11.4
TP [mgP L^−1^]	5.4	5.4	3.3	4.4
PO_4_–P [mgP L^−1^]	5.0	4.7	2.3	2.7
Sludge loading rate [kg totalCOD kgVSS^−1^ d^−1^]	0.26	0.21	0.26	0.26
Sludge volume index after 30 min (SVI_30_) [mL gTSS^−1^]^b^	43	51	84	65
Granule fraction d > 0.25 mm^b^	93%	63%	61%	74%
Typical granule diameter after 1 year^c^	1–3 mm	0.25–0.63 mm	0.25–0.63 mm	0.25–0.63 mm

a Detailed WW characterization can be found in Layer et al. (2019).

b Long-term average, not considering the start-up phase.

c Based on biomass size-fractions (Layer et al., 2019).

### 2.2 Modelling

#### 2.2.1 Model description

**Fig. 2:**
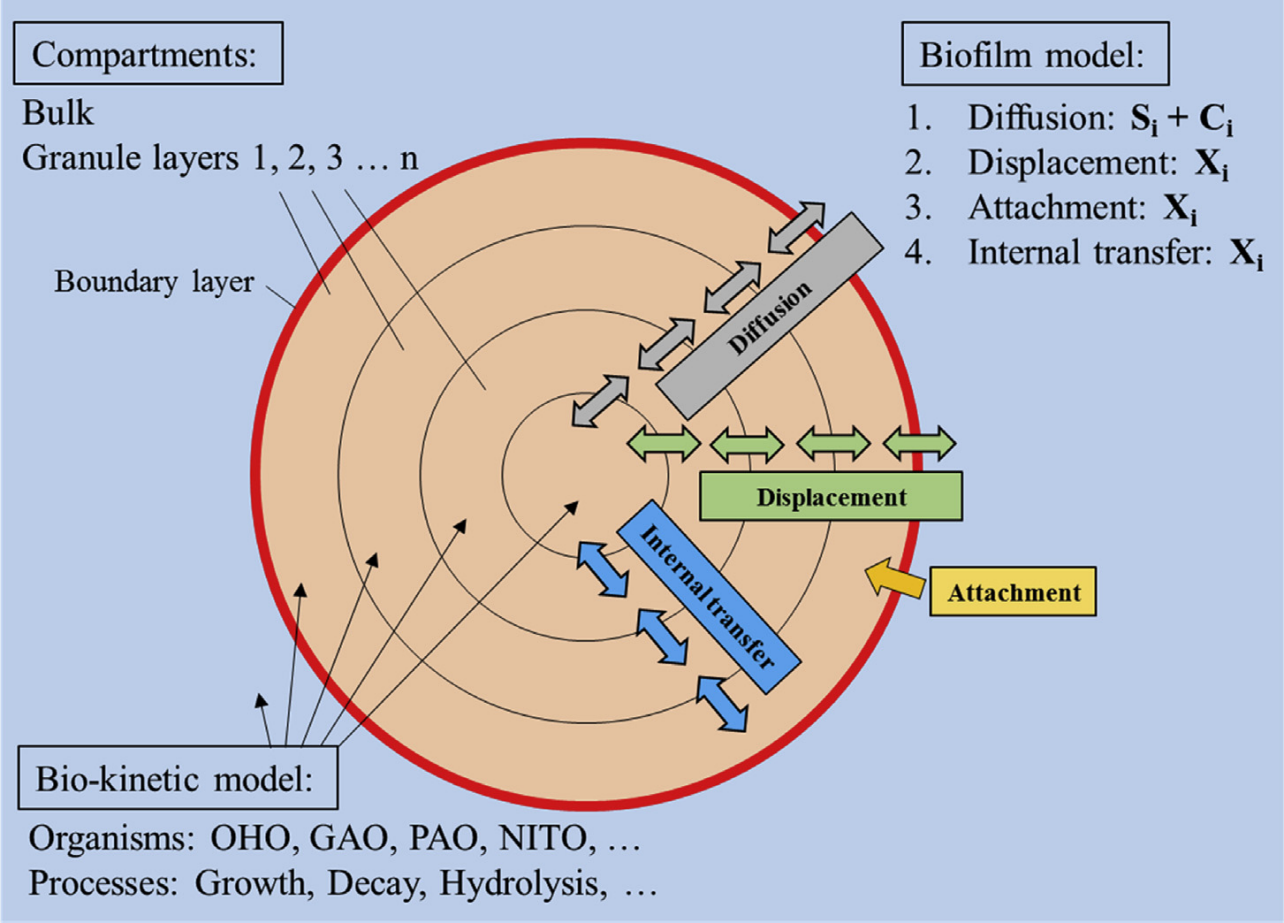
Conceptual model of the biofilm and biokinetic model used in this study. The compartments are composed of bulk and biofilm (granule) layers 1 to 10. The biofilm models mass balances based on the mass-transfer mechanisms (1) diffusion of soluble and colloidal compounds (S_i_ and C_i_), (2) displacement of X_i_, (3) attachment of Xi and (4) internal transfer of Xi. The biokinetic model is active in all compartments.

A mathematical model was used to identify the mechanisms governing SND in AGS systems, and test aeration strategies that optimise TN removal in AGS systems. The AGS model consists of (1) a biofilm model, (2) a biokinetic model, and (3) a reactor model (Fig. 2).

*Biofilm model:* The biofilm model is based on the 1-dimensional Wanner-Reichert mixed-culture biofilm model (Wanner and Reichert, 1996) implemented in SUMO® software (Version 16, Dynamita, France) (Dynamita, 2019). The main difference between the Wanner-Reichert and the SUMO biofilm models are that biofilm thickness is fixed (as an input) in the SUMO biofilm model, while it is predicted in the Wanner-Reichert model. In SUMO, the granule compartments are modelled as a sphere subdivided into n = 10 layers, and the individual layer area is calculated as a function of depth within the granule (Fig. 2). The total volume of granules is considered constant in the model. The biofilm model predicts mass-transfer processes between and within the fully mixed bulk phase and the different biofilm compartments (granule layers 1 to 10). The mass-transfer mechanisms implemented in the biofilm model are (1) diffusion of soluble and colloidal compounds (S_i_, C_i_) between all compartments (bulk and granule layers 1 to 10), (2) displacement of particulate compounds (X_i_), (3) attachment of X_i_ from the bulk to the granule surface layer and (4) internal transfer of X_i_ between the granule layers. Supplementary Information S2 provides detailed information on the biofilm model.

*Biokinetic model:* The biokinetic model used in this study (SUMO1, Varga et al., 2018) considers the following key microbial populations: ordinary heterotrophic organisms (OHO), glycogen accumulating organisms (GAO), phosphorus accumulating organisms (PAO) and nitrifying organisms (NITO). Nitrification and denitrification are modelled as 1-step processes. The decision is based on experimental evidences that both AOB and NOB are present in granules (Layer et al., 2019; Świątczak and Cydzik-Kwiatkowska, 2018; Ali et al., 2019; Winkler et al., 2012) so that short-cut nitrification-denitrification (nitrate shunt) is unlikely to occur (Figdore et al., 2018). Denitrification is performed by OHO, GAO and PAO. Denitrification by OHO is performed by using VFA or S_B_ (non-fermented readily biodegradable soluble substrate) as electron-donors. GAO can perform denitrification by using internally stored GLY (glycogen) while PAO perform denitrification using PHA (polyhydroxyalkanoates). OHO and PAO are additionally able to perform anaerobic fermentation of S_B_ to VFA. Supplementary Information Table S13 provides the complete biokinetic matrix and parameters.

*Reactor model:* The reactor model used in this study is an SBR. The SBR sequence set in the model was similar to the experimental one. All process steps were modelled in fully-mixed conditions. Effluent total suspended solids (TSS) were set to 20 mg L^−1^, in the range of the values measured experimentally (Layer et al., 2019). A solid retention time (SRT) of 20 d was maintained, in accordance to other AGS studies (De Kreuk et al., 2007b; Ni and Yu, 2010; Layer et al., 2019). SRT (d) was calculated based on Equ. (1).

(1) $$SRT_{target}=\;\frac{TSS_{r}\cdot V_{r}}{TSS_{eff}\cdot Q_{eff}+TSS_{bulk}\cdot Q_{ex}}$$

TSS_r_ is the TSS concentration in the reactor (gTSS L^−1^), V_r_ is the reactor volume (L), TSS_eff_ is the TSS concentration in the effluent (gTSS L^−1^), Q_eff_ is the effluent flow rate (L d^−1^), TSS_bulk_ is the TSS concentration in the bulk compartment (gTSS L^−1^) and Q_ex_ is the excess sludge flow rate (L d^−1^). Q_ex_ was automatically calculated based on SRT_target_ = 20 d. Excess sludge is only withdrawn from the bulk compartment. Supplementary Information S2 provides detailed information on the reactor model.

*Specific model adaptations:* Granules of diameters of 0.5 mm (young MWW granules) and 2.0 mm (mature VFA granules) were considered, in accordance with our experimental results collected over 1 year of operation (Table 1, Layer et al., 2019). Granule size of d = 2.0 mm for MWW (mature granules) was also selected in accordance with literature (Pronk et al., 2015). The individual layer thickness of the granule were set to 25 μm for all layers (d = 0.5 mm) or 25 μm for the 4 outer layers and 150 μm for the residual 6 inner granule layers (d = 2.0 mm). Decreasing the thickness of the 4 outer layers of the large granules increases the resolution in those layers, which is required to best predict the concentration gradients and redox conditions within the granules. For data plotting and interpretation, the distinction between aerobic, anoxic and anaerobic redox conditions is based on the half-saturation constants of growth on O_2_ and NO_x_ of OHO of the biokinetic model (Table 2).

**Table 2:** Conditions of DO and NO_x_-N that were defined as aerobic, anoxic and anaerobic conditions during modeling.

Redox condition	DO [mgO_2_ L^−1^]	NO_x_-N [mg NO_x_-N L^−1^]
Aerobic	≥0.05	–
Anoxic	<0.05	≥0.03
Anaerobic	<0.05	<0.03

#### 2.2.2 Modelling scenarios

Different modelling scenario were performed to (1) validate the overall performance of the model by comparing them to the batch test experiments (*batch test* scenarios), (2) understand the mechanisms of SND in AGS systems (*mechanism* scenarios), and (3) evaluate different optimised aeration strategies to maximize TN removal (*optimisation* scenarios) (Table 3).

**Table 3:** Overview of simulation scenarios and their corresponding granule diameters, biomass composition, aeration strategy and influent composition.

WW Type	*Batch test*scenarios		*Mechanism*scenarios		*Optimisation*scenarios	
	MWW	VFA	MWW	VFA	MWW	MWW
Granule diameter [mm]^a^	0.5	2.0	0.5/2.0	2.0	0.5/2.0	0.5/2.0
Aeration strategy	Constant DO	Constant DO	Constant DO	Constant DO	Alternating	2-step
COD [mg L^−1^]	469^b^	600 (as VFA)	500^b^	403 (as VFA)	500^b^	500^b^
TN [mg L^−1^]	41^b^	42 (as NH_4_–N)	41^b^	40 (as NH_4_–N)	41^b^	41^b^
TP [mg L^−1^]	4.4^b^	6 (as PO_4_–P)	5.1^b^	5.0 (as PO_4_–P)	5.1^b^	5.1^b^
Objective	validate the overall performance of the model by comparing them to the batch test experiments		better understand the mechanisms of SND in AGS systems		evaluate different optimised aeration strategies to maximize TN removal	

a as experimentally observed.

b with SUMO® standard WW fractionation, Supplementary Information Table S14.

The influent composition of the MWW cases was based on the “standard” fractionation provided by SUMO. The influent composition of the VFA cases were comprised of VFA as sole source of organic substrate, NH_4_^+^ as sole N source and PO_4_^3−^ as sole P source. See Supplementary Information Table S14 for detailed influent WW fractionation of MWW and VFA influent. All scenarios were comprised of a TSS of 5.9–6.6 gTSS L^−1^, and flocs represented 1–2% (VFA cases) to 7–9% of TSS (MWW cases), respectively. An overview of the relevant simulation parameters is given in Table 3 for the different scenario.

The “*batch test*” modelling-scenarios were performed to assess the ability of the model to correctly predict the experimental observations. The “*mechanism*” scenarios were performed to better understand the mechanisms influencing SND for different influent WW (MWW and VFA) and granule diameters (young and mature granules, d = 0.5 and 2.0 mm). Influent concentrations of 500 mg COD L^−1^, 41 mg N L^−1^ and 5.1 mg P L^−1^ were selected as the basis of MWW. The VFA WW consisted of the biodegradable fractions of COD, TN and TP of MWW and comprised 403 mg COD L^−1^, 40 mg N L^−1^ and 5.0 mg P L^−1^, respectively. For the different conditions, simulations were first run for 150 d at DO = 2.0 mg O_2_ L^−1^. DO was then changed, over two SBR cycles only, to different values of 0.5–6.0 mgO_2_ L^−1^. Data of the second cycle were then extracted. Matlab (Version R2018a, MathWorks, USA) was used for further data analysis, and SigmaPlot (Version 12.0, Systat, USA) was used for visualization of the data.

Finally, two different aeration strategies were tested to evaluate how to optimise the SND and TN removal during treatment of municipal WW with AGS (*optimisation* scenarios): alternating aeration (scenario #1) and 2-step aeration (scenario #2) (Table 3). For the “*alternating aeration*” scenario, the DO was switched on (DO = 2.0 mgO_2_ L^−1^) and off (DO = 0.0 mgO_2_ L^−1^) every 15 min during the aerobic SBR phase. For the “*2-step aeration*” scenario, the DO set-point was set at 2.0 mgO_2_ L^−1^ for 15 min, followed by 0.5 mgO_2_ L^−1^ for the residual 270 min of the aerobic SBR phase.

### 2.3 Calculations

The specific rate of ammonium removal (mgN gVSS^−1^ h^−1^) was calculated using Eq. (2).

(2) $$r_{NH_{4}-N}=\frac{c_{NH_{4}-N,\;start\_aerobic}-\;c_{NH_{4}-N,end\_nitrification}}{time_{nitrification}*VSS_{reactor}}$$

The specific rate of NO_x_-N accumulation (mgN gVSS^−1^ h^−1^) is calculated using Eq. (3). Equation (3) includes only the rate of NO_x_ produced simultaneously during nitrification.

(3) $$r_{NO_{x}-N}=\frac{c_{NO_{x}-N,\;end\_nitrification}-\;c_{NO_{x}-N,start\_aerobic}}{time_{nitrification}*VSS_{reactor}}$$

The SND efficiency (%) was calculated by dividing the amount of NO_x_ denitrified by the amount of NH_4_^+^ removed, according Eqs. (4), (5), (6), (7). These calculations neglect the contribution of N-assimilation to NH_4_^+^ or NO_x_ removal.

(4) $$SND=\frac{NO_{x}-N_{denitrified}}{NH_{4}-N_{removed}}$$

(5) $$\;NH_{4}-N_{removed}=c_{NH_{4}-N_{start\_aerobic}}-c_{NH_{4}-N_{end\_nitrification}}$$

(6) $$\;NO_{x}-N_{accumulated}=c_{NO_{x}-N_{end\_nitrification}}-c_{NO_{x}-N_{influent}}$$

(7) $$\;NO_{x}-N_{denitrified}=NH_{4}-N_{removed}-NO_{x}-N_{accumulated}$$

The aerobic, anoxic and anaerobic electron-donor utilisation rates (“*substrate utilisation rates*”) were extracted separately for each electron-donor (S_B_, VFA, GLY and PHA) directly from SUMO for each compartment (bulk and all granule layers). Supplementary Information Table S15 provides a detailed list of electron-donors utilisation by OHO, PAO and GAO in different redox conditions. The different volumes of individual granule layers were accounted for by normalization with the total reactor volume.

### 2.4 Analytical methods

Samples of influent and effluent during long-term experiments were analysed for total nitrogen (TN) using photochemical tests (Hach Lange, Germany, LCK 238 and 338). Cations (NH_4_-N) and anions (NO_2_−N, NO_3_−N) were analysed using flow injection analysis (Foss, FIAstar flow injection 5000 analyzer, Denmark) and anion chromatography (Methrom 881 compact IC, Switzerland), respectively.

## 3 Results

### 3.1 How is SND influenced by WW composition during long-term operation? (experimental results)

**Fig. 3:**
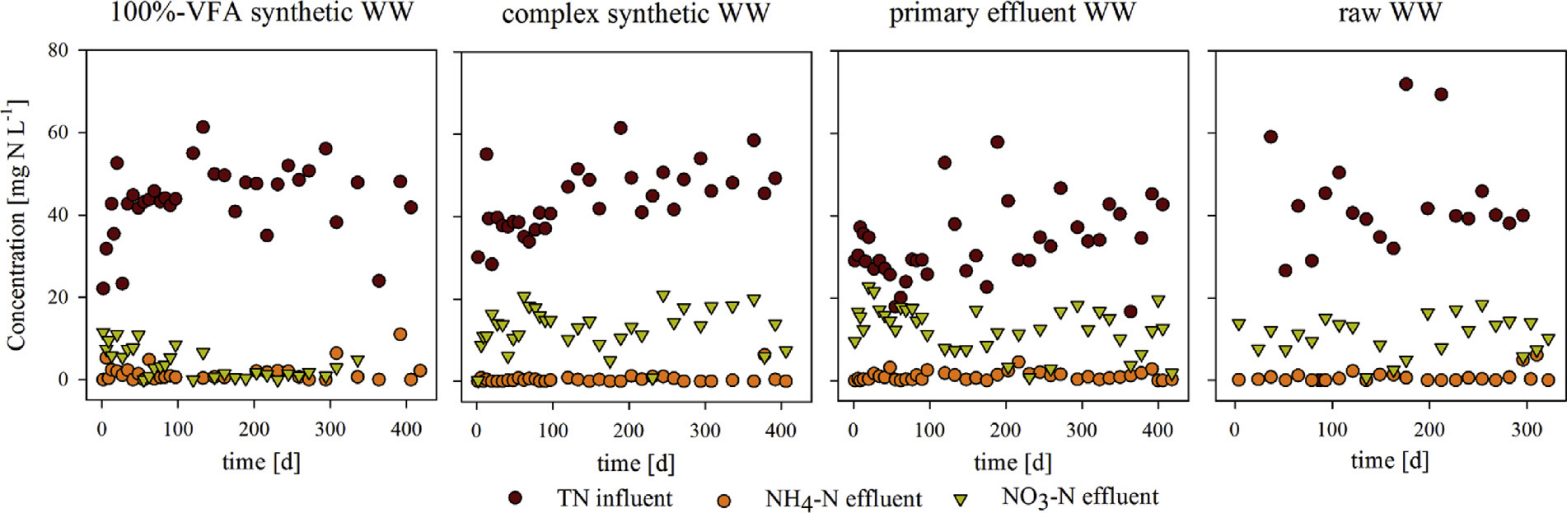
Long-term effluent NH_4_–N and NO_3_–N and influent TN concentrations for 100%-VFA synthetic, complex synthetic, primary effluent and raw WW fed AGS systems.

The nitrogen influent and effluent concentrations were monitored over the course of 300–400 days for the 4 reactors (Fig. 3). TN influent concentrations were similar for all reactors, ranging from 30 to 50 mg N L^−1^ on average. Also, low ammonium effluent concentrations <2 mg NH_4_–N L^−1^ were measured, indicating that full nitrification occurred in all reactors. In addition, NO_2_–N effluent concentrations were negligible in all reactors. The effluent NO_3_^-^ concentrations and thus the SND efficiencies were however significantly influenced by the influent composition. Average effluent NO_3_− concentrations below 4 mg NO_3_–N L^−1^ were measured for AGS system fed by 100%-VFA synthetic WW only, while values of 5–15 mg NO_3_–N L^−1^ were measured in the effluents of the complex synthetic, primary effluent and raw WW AGS systems, respectively.

### 3.2 How does DO concentration affect SND performance? (experimental and modelling results)

**Fig. 4:**
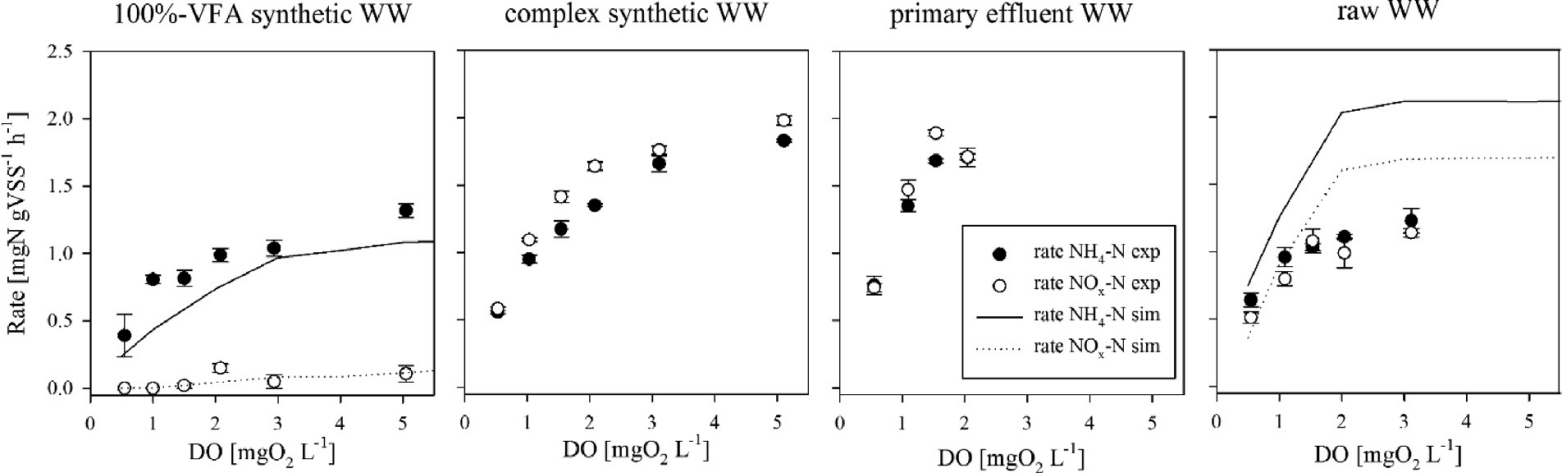
Change in the rates of NH_4_–N removal and NO_x_-N accumulation for AGS systems fed with different types of WW: 100%-VFA synthetic, complex synthetic, primary effluent and raw WW fed AGS systems during batch tests (rate NH_4_–N, NO_x_-N exp, indicated by dots), and model simulations of VFA and MWW (*batch test* scenarios*,* rate NH_4_–N, NO_x_-N sim, indicated by lines).

Batch tests and model simulations were performed at different bulk DO concentrations to confirm the effect of the influent composition in terms of simple vs. complex electron-donor composition on SND (Fig. 4). Results from batch-tests confirmed observations made over long-term operation of the 4 reactors, *i.e.*, that SND is strongly influenced by the influent composition in terms of organic substrate. Low SND, characterized by high NO_x_ accumulation rates, was observed for AGS systems treating complex WW (complex synthetic + real municipal WW). On the contrary, high SND was observed for AGS fed with 100%-VFA synthetic WW, independent from the DO concentration maintained in bulk (Fig. 4).

NH_4_^+^ removal rates increase with an increasing bulk DO concentrations for all tested WW conditions. Maximum NH_4_^+^ removal rates of 2 mg NH_4_–N gVSS^−1^ h^−1^ were measured for bulk DO concentrations larger than 2 mg O_2_ L^−1^. The NO_x_ accumulation rates almost match the NH_4_^+^ removal rates for AGS systems fed with complex WW (complex synthetic, primary effluent and raw WW, Fig. 3), indicative of the absence of SND in these systems. High SND only occurred in the AGS systems treating 100%-VFA synthetic WW, as indicated by the (very) low NO_x_ accumulation rates, *i.e.*, high denitrification rate.

Modelling results (*batch test* scenarios) correctly matched experimental observations of NH_4_^+^ removal- and NO_x_ accumulation rates – and thus SND performance - with changing DO (Fig. 4, plain line). Low NO_x_ accumulation rates (high SND) were predicted by the model for AGS fed with VFA WW, while high NO_x_ accumulation rates (low SND) were predicted for AGS fed by MWW. NH_4_^+^ removal rates increased for increasing DO bulk concentrations, and overall higher NH_4_ removal rates were observed for young AGS (d = 0.5 mm) fed with MWW, in comparison to AGS fed with VFA WW (d = 2.0 mm).

### 3.3 Dynamics of redox zone formation (modelling results)

**Fig. 5:**
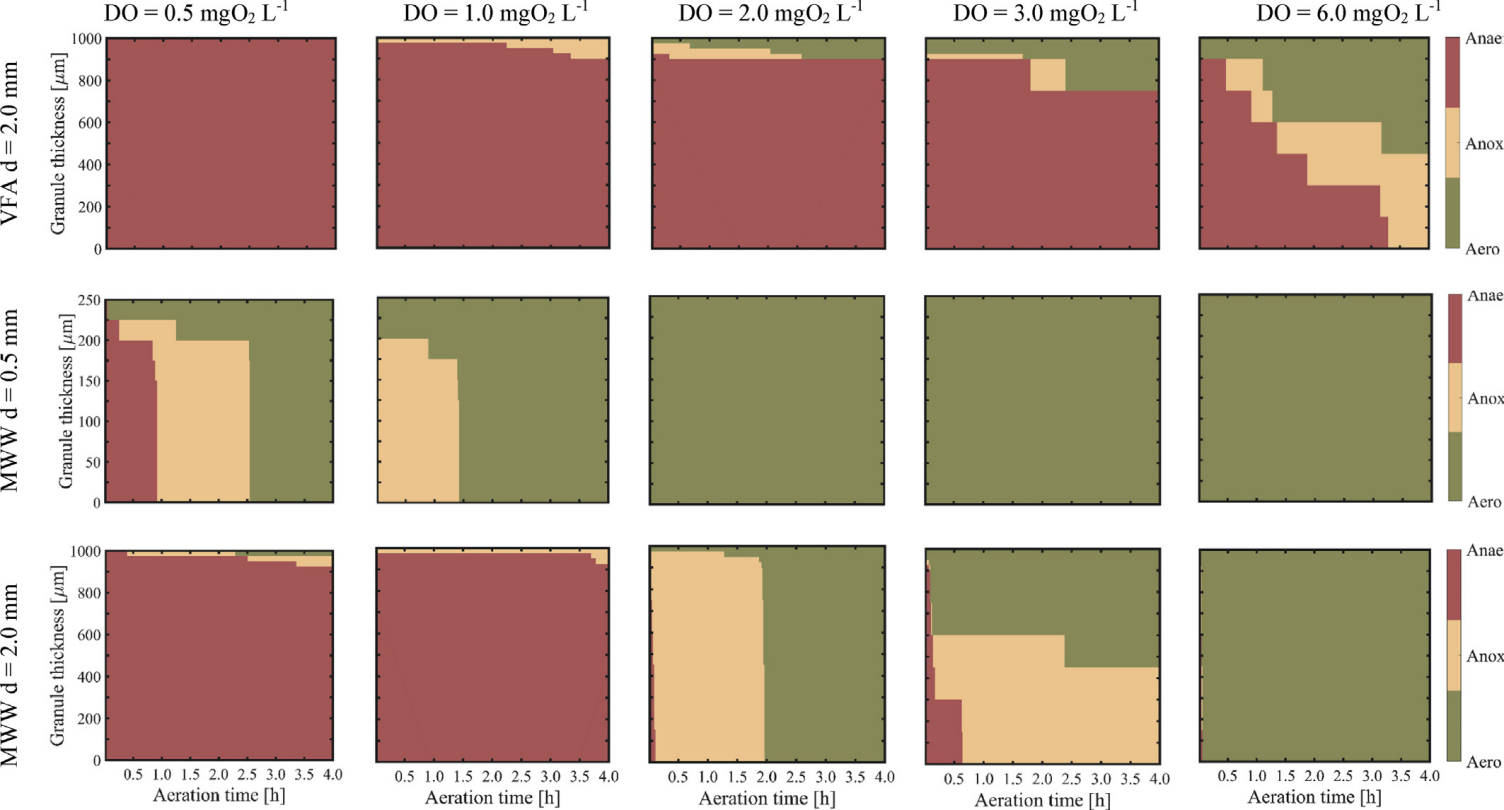
Effect of influent composition and granule diameter on dynamics of redox zone formation for different DO concentrations during the aerated phase in the bulk: 0.5, 1, 2, 3 and 6 mgO_2_ L^−1^ for VFA (granule diameter 2.0 mm) and MWW (granule diameters of 0.5 and 2.0 mm). Anaerobic zones are indicated in red (Anae), anoxic zones in yellow (Anox), aerobic zones in green (Aero). Granule thickness from core (0 μm) to the surface (250 or 1000 μm). (For interpretation of the references to colour in this figure legend, the reader is referred to the Web version of this article.)

The formation of the different redox zones was predicted during the aerobic phase for different DO concentration in the bulk (*mechanism* scenarios) (Fig. 5). The simulations indicate that (1) the formation of anoxic zones inside the granules depends on influent composition, granule size and bulk DO concentration and that (2) the formation of these anoxic zones is particularly dynamic, and thus significantly changes during the aerobic phase.

The composition of the influent WW governs the formation of the redox zones. Penetration depth of oxygen significantly reduces towards the deeper layers of the granule in case of VFA influent, compared to the MWW case with the same granule diameter (d = 2.0 mm) at the same DO concentration. In addition, the granule diameter governs how fast and deep oxygen penetrates the granule. Oxygen penetration is limited to 50–100 μm from the surface for AGS fed with VFA and DO = 2 mgO_2_ L^−1^ during the entire aerobic phase, while oxygen penetrates the entire granule immediately after aeration starts (d = 0.5 mm) or 2 h of aeration (d = 2.0 mm) for AGS fed by MWW. The third influencing factor affecting anoxic zone formation and dynamic is the DO concentration in the bulk. Increasing DO concentrations generally result in faster and deeper penetration of oxygen towards the deeper granule layers, independent of influent WW composition or granule size.

### 3.4 Which electron donors are actually used for denitrification? (modelling results)

**Fig. 6:**
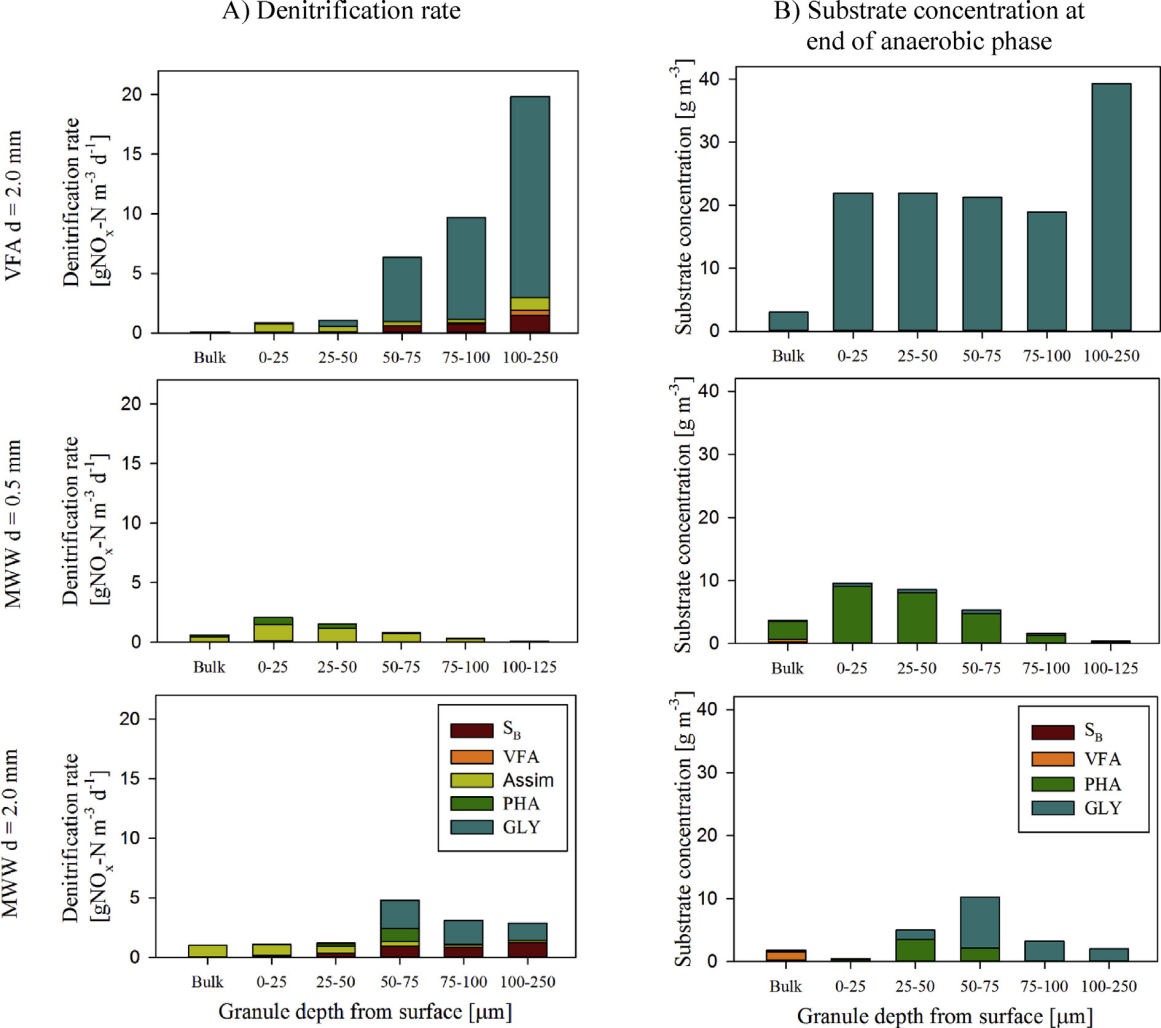
A) Contribution of the electron-donors S_B_, VFA, Assimilation of NO_x_ (Assim), PHA and GLY to the denitrification rate for VFA influent, granule diameter 2.0 mm (VFA d = 2.0 mm) and MWW influent, granule diameter 0.5 mm and 2.0 mm (MWW d = 0.5 mm and d = 2.0 mm) during the aerobic phase at constant aeration at 2 mgO_2_ L^−1^ displayed over the depth of the granule. B) Concentration of substrate (electron-donors) S_B_, VFA, PHA and GLY at the end of the anaerobic phase normalized to the reactor volume.

The denitrification rates were predicted for each electron-donor and in each layer of the granule during constant aeration at DO = 2.0 mgO_2_ L^−1^ (Fig. 6A). The concentration of each electron-donor available at the end of the anaerobic phase in each granule layer is also provided (Fig. 6B). The model predictions suggest that the higher the availability of electron-donor at the end of the anaerobic phase, the higher the denitrification during SND. The availability and utilisation of electron-donor via denitrification is highly influenced by the influent WW composition and granule diameter.

The denitrification rates predicted for AGS fed with VFA WW are 5–6 fold larger than those predicted for MWW influent with young granules (d = 0.5 mm) and 2–4 fold over the MWW influent with mature granules (d = 2.0 mm). For AGS systems fed with VFA WW, the main electron-donor for denitrification is glycogen (GLY). A minor fraction of NO_x_ is removed via assimilation or readily biodegradable S_B_. For small granules (d = 0.5 mm) fed with MWW, denitrification does not occur via GLY but rather through assimilation. Assimilation is the dominant pathway for TN removal for small granules fed with MWW. For large granules (d = 2.0 mm), the majority of denitrification is achieved utilising GLY as electron-donor, and to a smaller extent PHA and S_B_. Concentrations of internally stored electron-donors PHA and GLY after anaerobic conditions are a good proxy for their contribution to denitrification. Electron-donors are however only utilised in denitrification, if anoxic conditions also occur in those granule layers (cf. Figure 5, Figure 6AB).

### 3.5 Is there potential for electron-donor shift towards anoxic utilisation pathways? (modelling results)

**Fig. 7:**
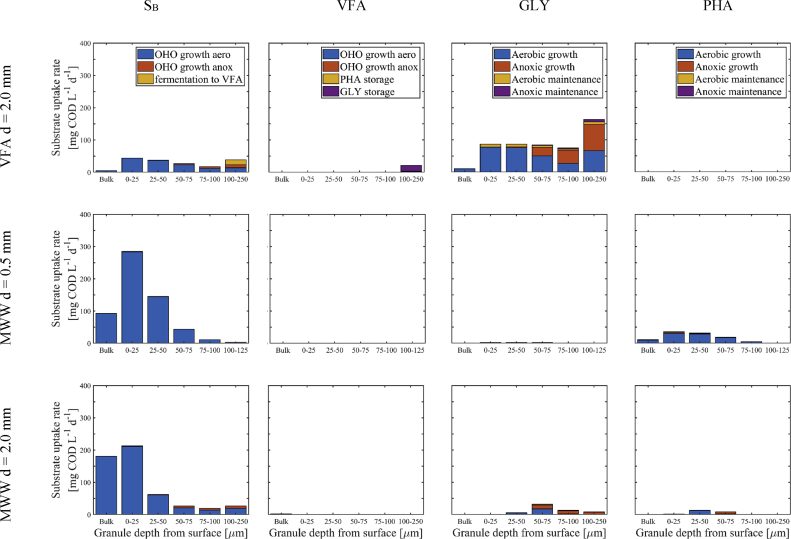
Uses of the electron donors S_B_, VFA, GLY and PHA during the aeration phase in the different biomass compartments. Only bulk and granule compartments (layers) 1–5 are displayed. Bulk phase DO was 2.0 mgO_2_ L^−1^.

Aerobic, anoxic and anaerobic utilisation pathways of the different electron donors (readily biodegradable (S_B_), VFA, glycogen (GLY) and PHA were analysed during the aerobic phase with constant aeration at DO = 2.0 mgO_2_ L^−1^ (Fig. 7). A main observation is that a major fraction of the electron-donors is utilised aerobically. Also, almost no electron-donors are utilised in anoxic conditions in AGS fed with MWW, while there is some anoxic utilisation in AGS fed with VFA.

Only VFA fed AGS gathered the prerequisites needed for significant anoxic utilisation of GLY in multiple granule layers, while readily biodegradable S_B_ is mostly utilised aerobically. No PHA utilisation – neither aerobic nor anoxic – is observed in VFA fed AGS, which is probably linked to competition with GLY. In MWW fed AGS on the other hand, aerobic growth is the main utilisation pathway for S_B_, VFA, GLY and PHA. Anoxic electron-donor utilisation is almost absent in MWW conditions for young granules (d = 0.5 mm) and only occurs to a minor extent in the inner granule layers via anoxic utilisation of GLY for mature granules (d = 2.0 mm). The model predictions indicate a large potential for shifting the utilisation of S_B_, PHA and GLY from aerobic to anoxic conditions and thus to increase SND and overall TN removal performances.

### 3.6 Can SND and TN removal be improved by optimising the aeration strategy? (modelling results)

**Fig. 8:**
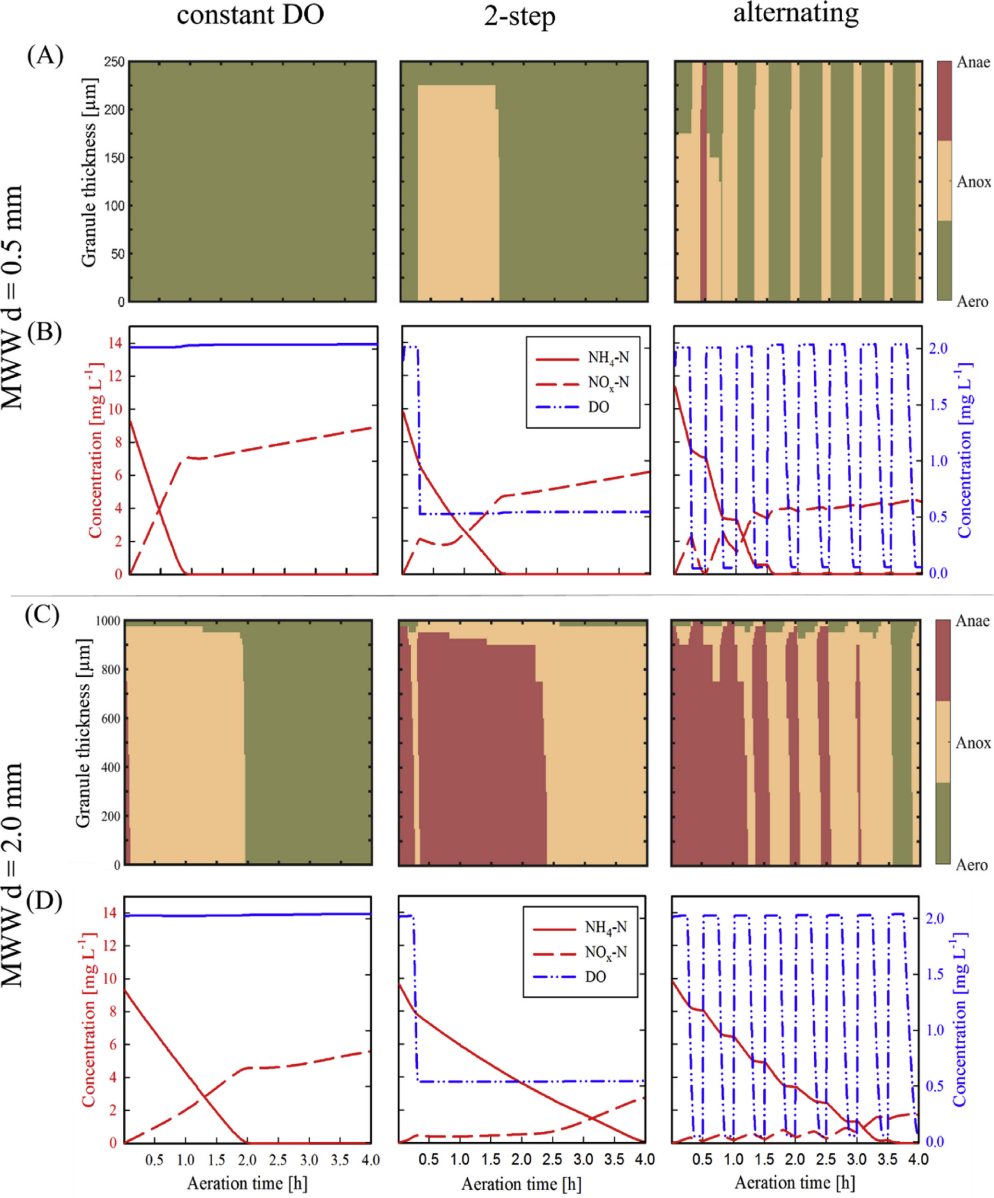
Redox-conditions and concentrations of NH_4_^+^, NO_x_ and DO during aerobic conditions of the simulated SBR cycle for MWW influent and young granules (d = 0.5 mm) (A,B) and mature granules (d = 2.0 mm) (C,D), for the three aeration strategies tested: constant DO, 2-step and alternating aeration.

**Fig. 9:**
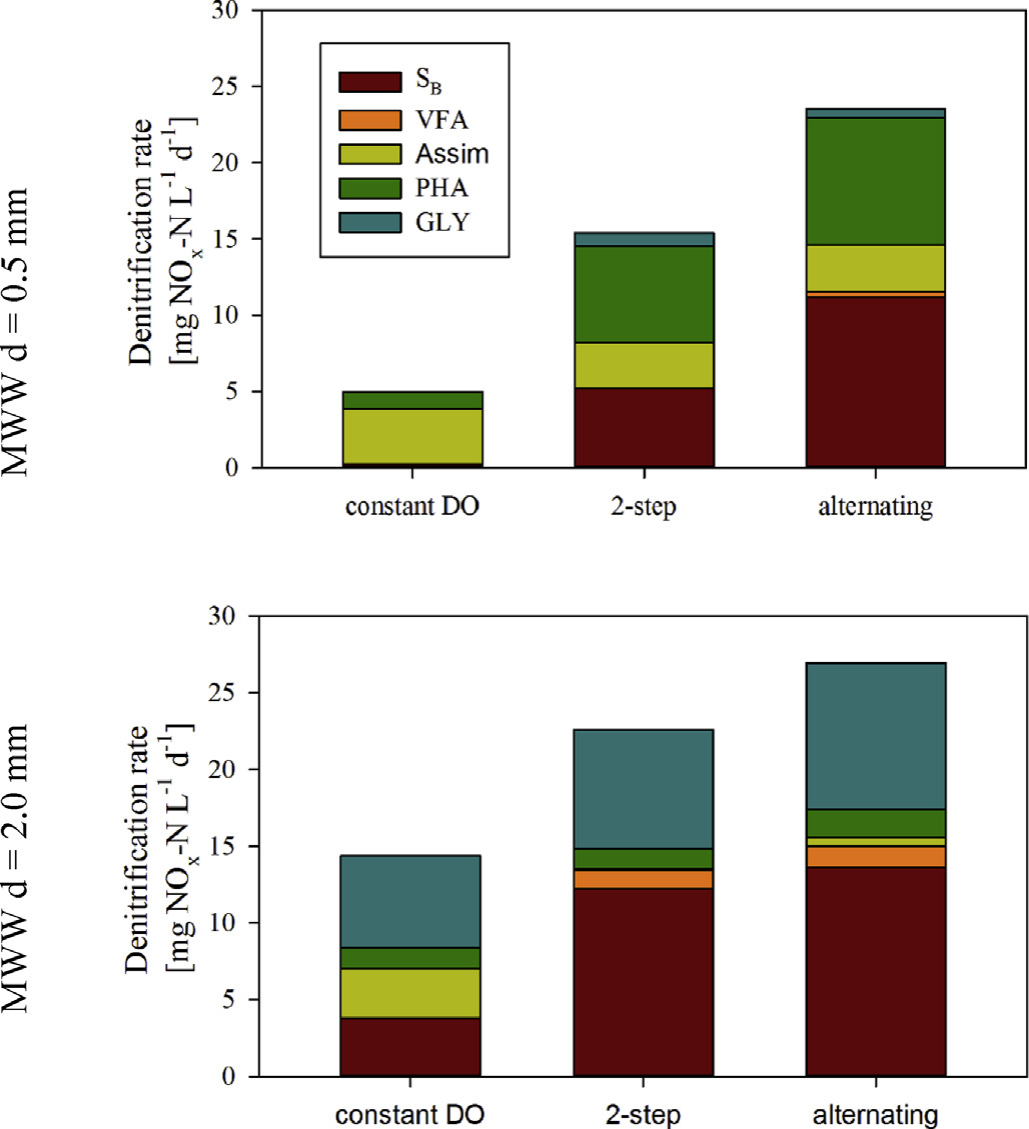
Contribution of the electron-donors S_B_, VFA, Assimilation of NO_x_ (Assim), PHA and GLY to the denitrification rate during aerobic conditions of the simulated SBR cycle for MWW influent, young granules (d = 0.5 mm) and mature granules (d = 2.0 mm) for the three aeration strategies tested: constant DO, 2-step and alternating aeration.

If most of S_B_, PHA and GLY is utilised via aerobic utilisation pathways, a main question is *to what extent can the aeration strategy be optimised to increase SND and ultimately TN removal?* The performance of alternating aeration and 2-step aeration were thus compared to the constant DO base case scenarios MWW d = 0.5 and 2.0 mm in terms of nitrification and denitrification performance (Table 4, Fig. 8 and Fig. 9). Applying an optimised aeration help to significantly increase denitrification efficiencies, while full nitrification was maintained for both young (d = 0.5 mm) and mature granules (d = 2.0 mm).

**Table 4:** Treatment efficiencies of different aeration strategies for otherwise similar operation of AGS SBR fed by MWW and granule diameters 0.5 and 2.0 mm.

Scenario	Aeration strategy	DO setpoint	Nitrification efficiency [%]	Denitrification efficiency [%]	Main NO_x_-N removal mechanism (% of total NO_x_-N removal)
MWW d = 0.5 mm	constant DO	Constant at 2 mg_O2_ L^−1^	99.9	13.6	Assimilation (72%)
	2-step	2-DO setpoint: 2 and then 0.5 mg_O2_ L^−1^	99.9	39.7	PAO using PHA (40%)
	alternating	Alternating between 2 and 0 mg_O2_ L^−1^	99.9	60.9	OHO using S_B_ (47%)
MWW d = 2.0 mm	constant DO	Constant at 2 mg_O2_ L^−1^	99.9	38.9	GAO using GLY (42%)
	2-step	2-DO setpoint: 2 and then 0.5 mg_O2_ L^−1^	99.7	67.3	OHO using S_B_ (54%)
	alternating	Intermittent between 2 and 0 mg_O2_ L^−1^	99.9	79.2	OHO using S_B_ (51%)

Denitrification efficiencies were increased by a factor of 2–4 to 40–61% for young (d = 0.5 mm) and by a factor of 1.5–2 to 65–79% for mature (d = 2.0 mm) granules, compared to 14–39% for AGS composed of young and mature granules operated at constant DO aeration, respectively. Despite the high increase in the SND efficiencies, optimising the aeration strategy does not allow achieving full denitrification. Optimised aeration helps to better control the formation of anoxic zones within the granule, and in turn the anoxic electron-donor utilisation (Fig. 8A,C, Fig. 9). Granule size still influences the extent of anoxic zone formation: larger anoxic zones develop for a longer period for mature granules (d = 2.0 mm) than for young granules (d = 0.5 mm). In the case of young granules (d = 0.5 mm), the main contributor to denitrification shifted from assimilation in the constant DO base case (due to absence of anoxic redox zones) to S_B_ and PHA in the optimisation scenarios (Fig. 9). For mature granules (d = 2.0 mm), the main electron-donor for denitrification shifted from glycogen (GLY) in constant DO aeration to S_B_ in the optimisation scenarios, and the contribution of assimilation to SND decreased to almost zero.

## 4 Discussion

### 4.1 SND and TN removal is limited in AGS systems treating municipal WW

It is usually well recognised that high TN removal via SND is a key attribute of AGS systems (Chen et al., 2011; De Kreuk et al., 2005; He et al., 2017). However, our long-term experiments indicated that limited SND is observed for AGS systems treating municipal WW, while full TN removal via SND is representative of systems fed with VFA only. Those results are supported by both experiments and mathematical modelling (Fig. 3, Fig. 4, Fig. 6). Experimentally, only AGS fed with VFA achieved high SND and NO_3_^−^ effluent concentrations below 4 mg NO_3_–N L^−1^. Effluent NO_3_–N larger than 10 mg NO_3_–N L^−1^ were on the other hand measured for AGS treating municipal WW in our experiments. Other studies indicate a similar trend. Limited SND was previously reported for AGS treating municipal WW (Derlon et al., 2016; Wagner et al., 2015) and effluent NO_3_–N of 2–6 mg NO_3_–N L^−1^ and TN effluent concentrations of 5–15 mg N L^−1^ were measured for a Nereda® full-scale plant (Pronk et al., 2015). But in the study of Pronk et al. (2015), the majority of denitrification actually occurs once the DO was reduced to 0.5 mg L^−1^ (Supplementary Information S1). Our modelling results indicate a similar range of SND efficiencies. During constant DO aeration only 14–39% of SND efficiency were achieved by AGS composed of small and large granules in municipal WW conditions, respectively. Low SND efficiency results in high effluent TN concentrations without application of specific measures. Conversely, VFA fed AGS achieved SND of 90% in our simulations. Thus, one should not expect full TN removal via SND in the case of AGS system treating municipal WW and operated at constant DO. A main aspect is to discuss how the WW composition influence the SND and how to increase the efficiency of AGS system treating municipal WW.

### 4.2 What mechanisms limit SND in AGS systems treating municipal WW?

Low SND efficiency in AGS systems treating municipal WW is mainly governed by (1) the formation of limited anoxic zones inside the granule and (2) simultaneous lack of electron-donor available for denitrification. Concurrently, nitrification is not limiting SND.

Penetration of oxygen is one of the key factors influencing denitrification inside granules or biofilms in general (Nielsen et al., 1990). Our results demonstrate that, as a result of the SBR mode, the formation of anoxic conditions within the granules is a very dynamic process. A too fast and deep penetration of oxygen inside the granules limits the formation of anoxic zones and thus denitrification (Hibiya et al., 2004). On the contrary, if oxygen availability is too low, nitrification is then limited. When DO is controlled at a constant value of 2 mg L^−1^, the entire granule volume is anaerobic during the first minutes of aeration, due to the simultaneous absence of oxygen (quickly consumed due to high microbial activities) and of NO_x_ (nitrification has not started yet). After few minutes, NO_x_ are then produced by nitrification and diffuse through the granules, resulting in the formation of anoxic conditions towards the granule core. Oxygen gradually penetrates deeper inside the granules as substrates get converted (reduced oxygen uptake rate at the surface). This deeper penetration of oxygen in turn reduces the anoxic zone. Increasing bulk DO thus results in (1) a faster appearance of anoxic zones inside the granules at the beginning of the aerobic phase, but also (2) a faster disappearance of those anoxic zones at the end of the aerobic phase. Our modelling and batch experiment results confirm that SND can be greatly influenced by the DO applied in the bulk phase of the reactor (Mosquera-Corral et al., 2005; He et al., 2019; Third et al., 2003). Granule size is another important determining factor of anoxic zone formation (Chen et al., 2011). Hereby, larger granules sustain anoxic zones for longer time. Granule size is important since it governs the volume and persistence of anoxic zones inside the granule, and larger diameters prevent from too fast and deep oxygen penetration through diffusion (Li et al., 2008). A main challenge is in finding a fine balance in terms of aeration, in order to achieve full nitrification and to establish anoxic zones in the core of the granules.

In addition to the dynamics of anoxic zone formation, the diffusion of electron-donors also strongly limit denitrification in AGS systems (Derlon et al., 2016). Only diffusible carbon sources, like VFA or S_B_, can reach the deeper layers of the granule, in which anoxic conditions are more likely to occur. VFA can be utilised directly or be stored as GLY or PHA during the prior anaerobic phase by GAO and PAO, respectively. The availability of VFA in the deep layers of the granule during the anaerobic SBR phase is crucial for subsequent denitrification during the aerobic SBR phase. In the case of municipal WW the lack of VFA (<5–10% of total COD) in the influent WW thus strongly limits SND. Readily biodegradable substrate S_B_ therefore becomes the most important electron-donor for denitrification in municipal WW conditions. Ideally, most of the influent S_B_ is stored as intracellular polymers (GLY and PHA) under anaerobic conditions, in order to favour granule formation. But aerobic utilisation of S_B_ can occur when part of S_B_ “leaks” into the aerobic phase, or is produced by hydrolysis of X_B_. The presence of readily biodegradable S_B_ in the aerobic SBR phase can benefit the growth of OHO. S_B_ can be used by OHO for denitrification if it is available in anoxic zones of the granule. The presence of OHO at the granule surface layers might also promote the establishment of strong oxygen gradients within the granule, which ultimately helps forming anoxic conditions inside the granule. However, a higher availability of S_B_ during the aerobic SBR phase can also be detrimental to the formation of granules, as this promotes aerobic growth of OHO. OHO in turn outcompete GAO and PAO. PAO/GAO favour granule formation, overall process stability and are essential for nutrient removal in AGS systems (De Kreuk and Van Loosdrecht, 2004). Another main outcome of our study is about the role of GAO. GAO are usually considered detrimental in EBPR systems, as they can hamper the biological phosphorus removal (Majed et al., 2012). However, their presence can be beneficial for TN removal, as suggested by our simulations and literature (Weissbrodt et al., 2013).

In the case of AGS fed with VFA WW, the overall electron-donor utilisation in denitrification is strongly shifted towards internally stored GLY by GAO. Thus, storage compounds such as GLY or PHA become the major electron-donor source during SND in such systems, depending on the presence of GAO/PAO. The absence of denitrification on PHA in the VFA influent AGS case can be explained by the high COD/P ratio in the influent, which promotes the growth of GAO over PAO (Majed and Gu, 2019).

Our results thus demonstrate that different mechanisms govern SND, depending on the influent WW composition and granule diameter. But in the case of municipal WW, our results indicate that the main electron-donor utilisation pathways of S_B_, VFA, GLY and PHA are almost exclusively aerobic. If most of the electron-donors are used aerobically during the aerobic phase operated at constant DO, optimised aeration strategies might help to direct electron-donors towards anoxic utilisation.

### 4.3 How to optimise for TN removal in AGS systems?

Our results indicate that large granule diameter is one of the parameters that promotes anoxic zone formation and thus the capability of the system to achieve high SND efficiency. However, in practice, controlling the granule diameter is very challenging as granule size is influenced by the granules age and the organic loading (Layer et al., 2019). Large granule diameters have been reported for full-scale AGS systems, but rather after few years of operation and during treatment of WW with a high readily biodegradable S_B_ content (Pronk et al., 2015). For this reason, improving SND via engineering of the granule size does not represent a relevant approach for optimising TN removal. Inoculation with large granules could impair start-up and denitrification performance, since SND could occur from the start of the system. Nevertheless, the final extent of SND remains strongly hampered by the low availability of diffusible electron-donors in municipal WW. Therefore, other approaches must be considered to improve the TN removal. Pre- or post-denitrification are typical options to increase TN removal in conventional activated sludge SBR systems or in AGS systems (Pronk et al., 2015). However, both come with drawbacks. Pre-denitrification does not prevent from high TN effluent concentrations and post-denitrification is associated with very low rates, and hence SBR cycle duration must be increased.

Our results demonstrate that optimising the aeration strategy represents a simple and efficient approach to improve the TN removal of AGS systems. An increase from 14-37% (constant DO aeration) to 65–79% (2-step and alternating aeration) of the denitrification efficiency could be achieved. The increase in the denitrification efficiency results from an improved utilisation of the electron-donors under anoxic conditions. In previous studies, optimised aeration strategies for AGS systems have successfully increased TN removal too, like *e.g.* mathematical modelling of 2-step aeration (Sun et al., 2019), adaptable DO setpoint operation (Isanta et al., 2013), or lab-scale experiments on alternating aeration (Lochmatter et al., 2013). On full-scale installations, a 2-step aeration strategy is applied to overcome limited TN removal (Pronk et al., 2015; Supplementary Information S1). A recent patent on the aeration strategy of Nereda® reactors also confirms that specific measures must be taken to increase SND in AGS systems (Derlon et al., 2016; Layer et al., 2019), *i.e..*, to control the aeration rate based on a targeted NO_x_ concentration (Van Dijk et al., 2018). However, those studies lack fundamental understanding on anoxic zone formation or electron-donor utilisation affecting TN removal.

Our simulation results indicate that anoxic zone formation is much better controlled and prolonged when a 2-step and or an alternating aeration is applied. Especially, we observed that the electron-donor utilisation can be shifted, from mostly aerobic to anoxic. Significantly more S_B_ was thus utilised via anoxic pathways, and the contribution of assimilation to TN removal was almost zero, in comparison to constant DO aeration. In the case of large granules, VFA could be produced via fermentation and utilised inside the granule, additionally contributing to denitrification. Both effects result in a much higher denitrification efficiency in comparison to constant DO aeration. Therefore, high fractions and concentrations of diffusible electron-donors are not only favourable to improve start-up time and settling performance of AGS systems, but also SND performance (Layer et al., 2019). Limitations in TN removal due to unfavourable influent WW conditions can be overcome by optimised aeration strategies. The microbial pathways of electron-donor utilisation can directly be influenced and engineered by application of different aeration strategies.

However, drawbacks of the proposed aeration strategies to increase TN removal also exist. During 2-step and alternating aeration, a balance must be found between the high DO period (to achieve full nitrification) and low DO period (to maximize denitrification). Transient DO conditions – present in both optimised aeration strategies - were reported to trigger growth of filamentous bacteria, or breakage of granules (Martins et al., 2004; Sturm et al., 2004), or increased NO_2_^−^ formation during nitrification (Alleman, 1985). Accumulation of NO_2_^−^ ultimately increases the risk of N-losses via N_2_O production during nitrification/denitrification (Law et al., 2012). N_2_O has the potential to be the main greenhouse gas emission during WW treatment (Gruber et al., 2019). Therefore, N_2_O production during nitrification and denitrification should be avoided.

## 5 Conclusions

• Limited SND and TN removal is observed in AGS systems treating low-strength municipal wastewaters, thus resulting in high NO_x_ and TN effluent concentrations. Denitrification, not nitrification, is limiting SND in AGS systems.
• SND is limited by the very dynamic formation of anoxic zones and the availability of electron-donors within the granules. Anoxic zones only develop during a short period in a small volume of the granule. Larger granules and lower bulk DO concentrations prolong anoxic zones inside the granules and hence increase SND.
• The mechanisms and extent of anoxic substrate conversion at constant DO operation is governed by the composition of the influent WW (municipal or VFA-only WW). Internal storage compounds PHA and glycogen accounted for 40% (AGS municipal WW) or > 90% (AGS VFA-only WW) of electron-donors used in denitrification at constant DO operation. In municipal WW fed AGS systems, S_B_ is an important electron-donor in denitrification, too.
• Aeration strategies must be optimised to increase SND and TN removal during treatment of low-strength municipal WW using AGS systems. Alternating and 2-step aeration strategies help to increase TN removal from 13% to more than 65% during treatment of municipal low-strength WW.

## Declaration of competing interest

The authors declare that they have no known competing financial interests or personal relationships that could have appeared to influence the work reported in this paper.
