# Supplementary material for: Limited simultaneous nitrification-denitrification (SND) in aerobic granular sludge systems treating municipal wastewater: Mechanisms and practical implications
Source: Water Res X. 2020 Feb 27;7:100048. doi: 10.1016/j.wroa.2020.100048 (PMC7058409; doi:10.1016/j.wroa.2020.100048)
Supplement: Multimedia component 1 [file mmc1.docx]

Limited simultaneous nitrification-denitrification (SND) in aerobic granular sludge systems treating municipal wastewater: Mechanisms and practical implications

**SUPPLEMENTARY INFORMATION**

Manuel Layer^1^, Mercedes Garcia Villodres^1^, Antonio Hernandez^1^, Eva Reynaert^1^, Eberhard Morgenroth^1,2^, Nicolas Derlon^1^

*^1^ Eawag: Swiss Federal Institute of Aquatic Science and Technology, Überlandstrasse 133,*

*CH-8600 Dübendorf, Switzerland*

*^2^ Institute of Environmental Engineering, ETH Zürich, CH-8093 Zürich, Switzerland*

Email of the first author: manuel.layer@eawag.ch

Corresponding author: Nicolas Derlon (nicolas.derlon@eawag.ch)

# S1 SND efficiency calculations from literature

SND was calculated using Eq. (4) in the manuscript, and neglected the effect of assimilation on both NH_4_-N and NO_3_-N / NO_x_-N removal.


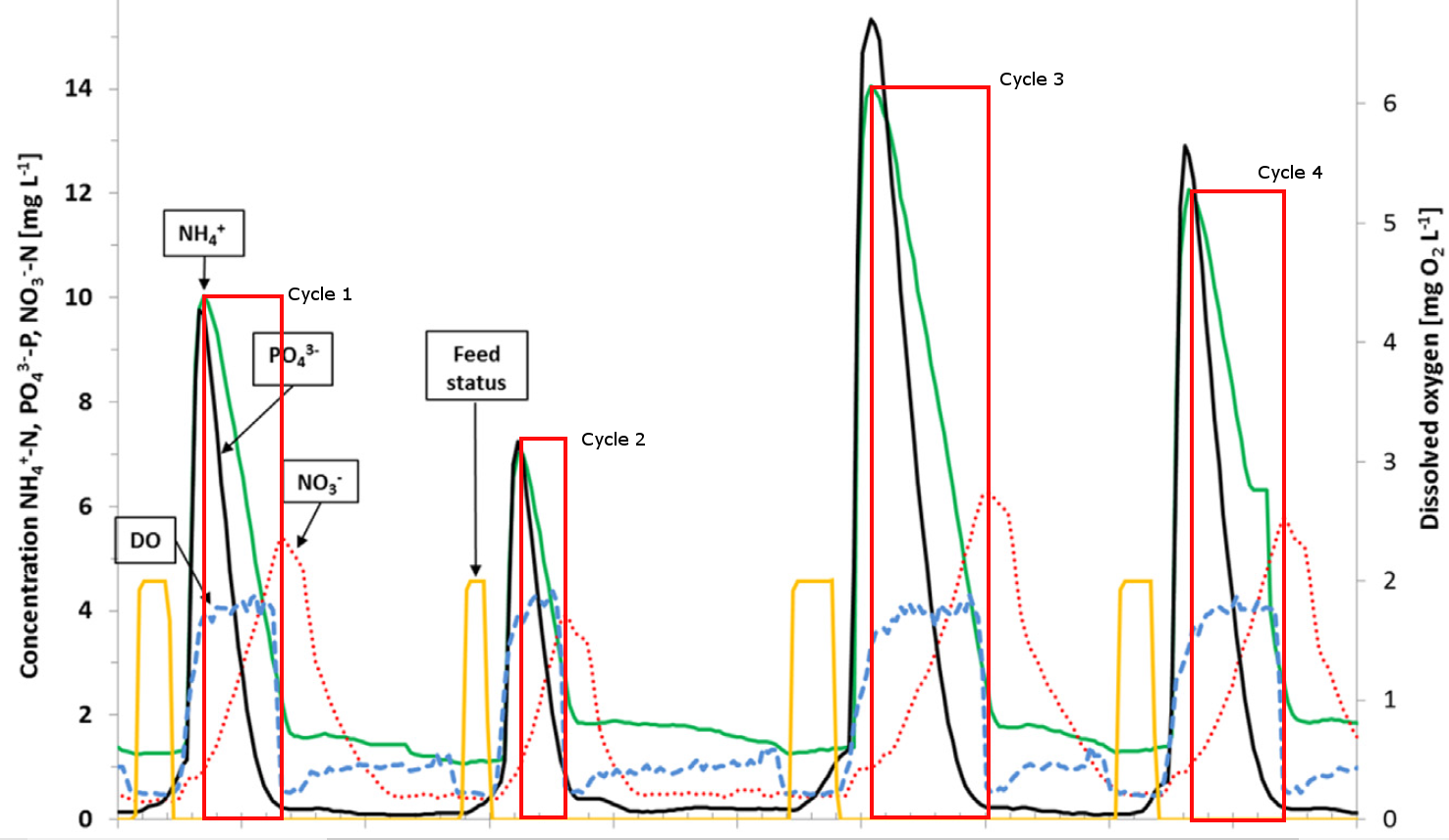


**Figure S2**: Figure 6 from Pronk et al., 2015 showing the concentration profiles of various compounds during 4 consequent SBR cycles. The red boxes marked with Cycle 1-4 indicate which parts of the concentration profiles of NH_4_-N and NO_3_-N were used to calculate SND during aeration at DO = 1.8 – 2.0 mgO_2_ L^-1^. After aeration at DO 2.0 mgO_2_ L^-1^, and after nitrification is finished, it can be seen that DO is controlled at 0.5 mgO_2_ L^-1^ for the residual SBR phase.

**Table S2:** SND calculations for concentration profiles from cycles 1-4 displayed in Figure S3. Plot Digitizer 2.6.8 was used to extract data from the figure Huwaldt, 2015.

| Cycle | NH_4_-N  start | NH_4_-N  end | NO_3_-N  start | NO_3_-N  end | | NH_4_-N  removed | | NO_3_-N accumulated | N  denitrified | SND |
| --- | --- | --- | --- | --- | --- | --- | --- | --- | --- | --- |
|  | mgN L^-1^ | mgN L^-1^ | mgN L^-1^ | | mgN L^-1^ | | mgN L^-1^ | mgN L^-1^ | mgN L^-1^ | % |
| 1 | 10.0 | 1.7 | 0.3 | 5.3 | | 8.4 | | 5.0 | 3.3 | 40% |
| 2 | 7.1 | 1.8 | 0.3 | 3.9 | | 5.3 | | 3.6 | 1.7 | 32% |
| 3 | 14.0 | 1.8 | 0.4 | 6.2 | | 12.2 | | 5.8 | 6.5 | 53% |
| 4 | 12.0 | 1.8 | 0.5 | 5.7 | | 10.2 | | 5.3 | 4.9 | 48% |


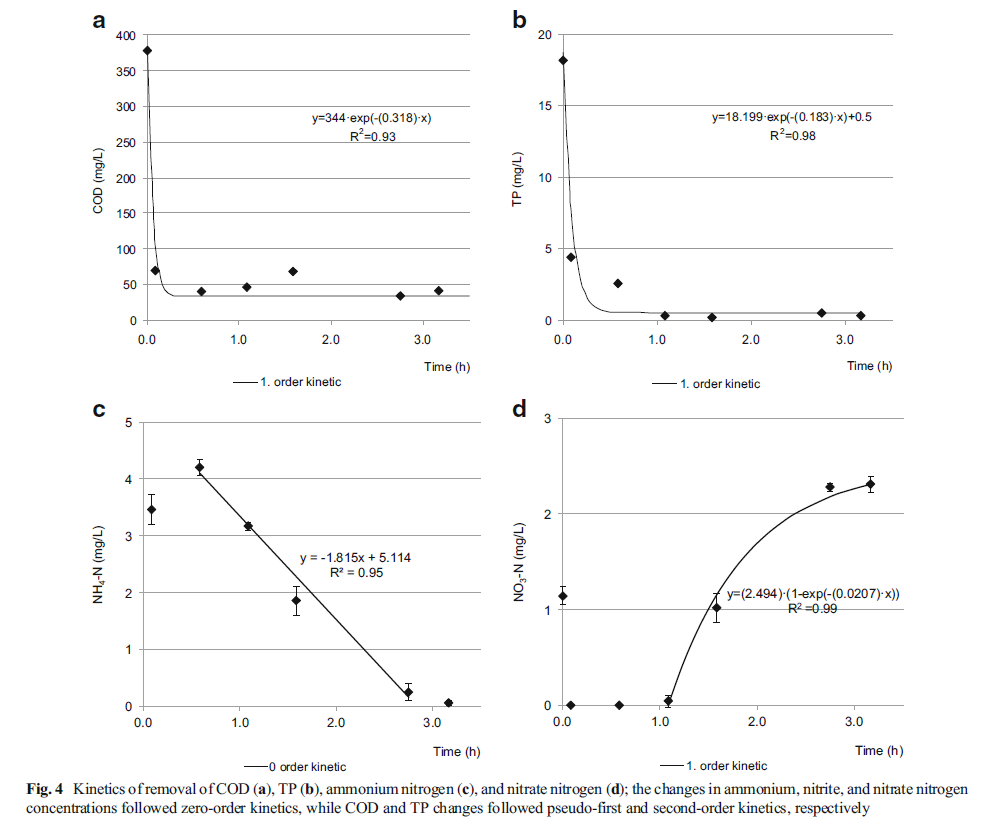


**Figure S3:** Figure 4 from Świątczak and Cydzik-Kwiatkowska, 2018 showing the concentration profiles of various compounds during one SBR cycle. The corresponding DO was 2.0 mgO_2_ L^-1^.

**Table S3**: SND calculations for the SBR cycle shown in Figure S4. Plot Digitizer 2.6.8 was used to extract data from the figure Huwaldt, 2015.

| NH_4_-N start | NH_4_-N end | NO_3_-N start | NO_3_-N end | NH_4_-N removed | NO_3_-N accumulated | N denitrified | SND |
| --- | --- | --- | --- | --- | --- | --- | --- |
| mgN L^-1^ | mgN L^-1^ | mgN L^-1^ | mgN L^-1^ | mgN L^-1^ | mgN L^-1^ | mgN L^-1^ | % |
| 4.21 | 0.07 | 0 | 2.33 | 4.14 | 2.33 | 1.81 | 43.7 |


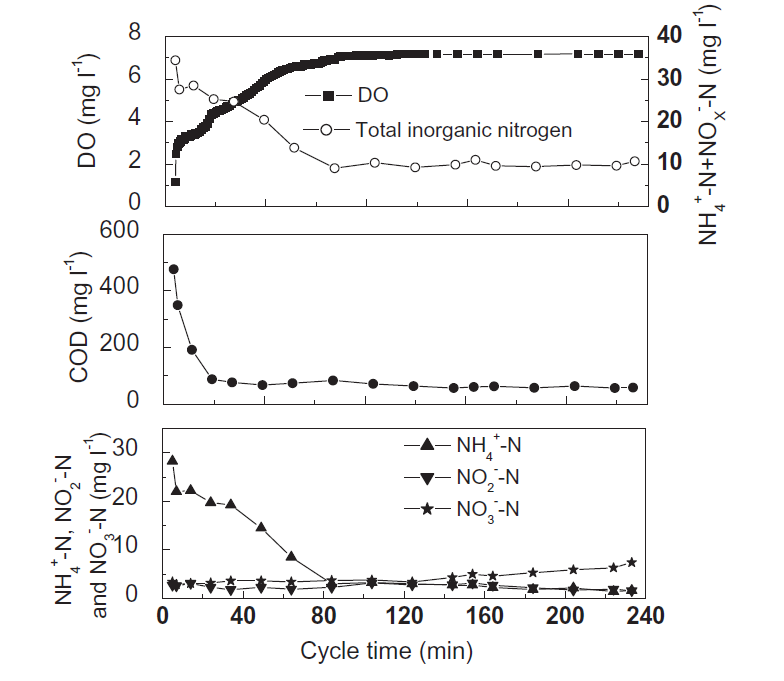


**Figure S4:** Figure 5 from Liu et al., 2011 showing the concentration profiles of various compounds during one SBR cycle. The corresponding DO was 7.16 mgO_2_ L^-1^.

**Table S4:** SND calculations for the SBR cycle shown in Figure S5. Plot Digitizer 2.6.8 was used to extract data from the figure Huwaldt, 2015.

| NH_4_-N start | NH_4_-N end | NO_2_-N start | NO_2_-N end | NO_3_-N start | NO_3_-N end | NH_4_-N removed | NO_x_-N accumulated | N  denitrified | SND |
| --- | --- | --- | --- | --- | --- | --- | --- | --- | --- |
| mgN L^-1^ | mgN L^-1^ | mgN L^-1^ | mgN L^-1^ | mgN L^-1^ | mgN L^-1^ | mgN L^-1^ | mgN L^-1^ | mgN L^-1^ | % |
| 28.34 | 1.66 | 2.48 | 1.66 | 3.31 | 7.45 | 26.68 | 3.32 | 23.36 | 87.6 |


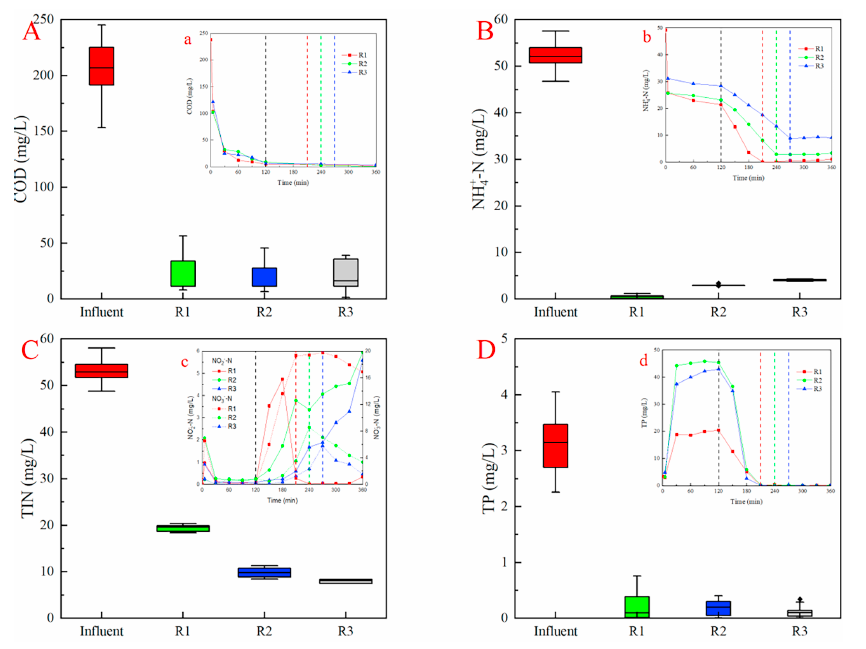
 **Figure Figure S5:** Figure 4 from He et al., 2019 showing the concentration profiles of various compounds during one SBR cycle. The corresponding DO were 7.75, 2.0 and 1.25 mgO_2_ L^-1^ for reactors 1, 2 and 3, respectively.

**Table S5:** SND calculations for the SBR cycle shown in Figure S6. Plot Digitizer 2.6.8 was used to extract data from the figure Huwaldt, 2015.

| Reactor | NH_4_-N influent | NH_4_-N effluent | TIN  influent | TIN  effluent | NH_4_-N removed | N denitrified | SND |
| --- | --- | --- | --- | --- | --- | --- | --- |
|  | mgN L^-1^ | mgN L^-1^ | mgN L^-1^ | mgN L^-1^ | mgN L^-1^ | mgN L^-1^ | % |
| 1 | 51.8 | 0.35 | 52.74 | 19.58 | 51.45 | 33.75 | 65.6 |
| 2 | 51.8 | 2.66 | 52.74 | 9.99 | 49.14 | 41.03 | 83.5 |
| 3 | 51.8 | 3.68 | 52.74 | 8.55 | 48.12 | 41.45 | 86.1 |


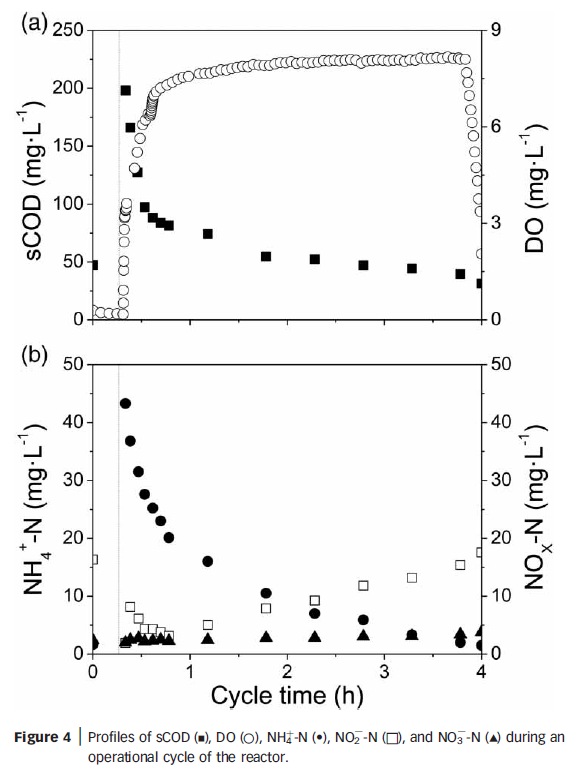

**Figure S6:** Figure 4 from Wagner et al., 2015 showing the concentration profiles of various compounds during one SBR cycle. The corresponding DO was 8.16 mgO_2_ L^-1^.

**Table S6**: SND calculations for the SBR cycle shown in Figure S7. Plot Digitizer 2.6.8 was used to extract data from the figure Huwaldt, 2015.

| NH_4_-N start | NH_4_-N end | NO_2_-N start | NO_2_-N end | NO_3_-N start | NO_3_-N end | NH_4_-N removed | NOx-N accumulated | N denitrified | SND |
| --- | --- | --- | --- | --- | --- | --- | --- | --- | --- |
| mgN L^-1^ | mgN L^-1^ | mgN L^-1^ | mgN L^-1^ | mgN L^-1^ | mgN L^-1^ | mgN L^-1^ | mgN L^-1^ | mgN L^-1^ | % |
| 43.45 | 1.72 | 8.28 | 17.76 | 2.07 | 3.97 | 41.73 | 11.38 | 30.35 | 72.7 |


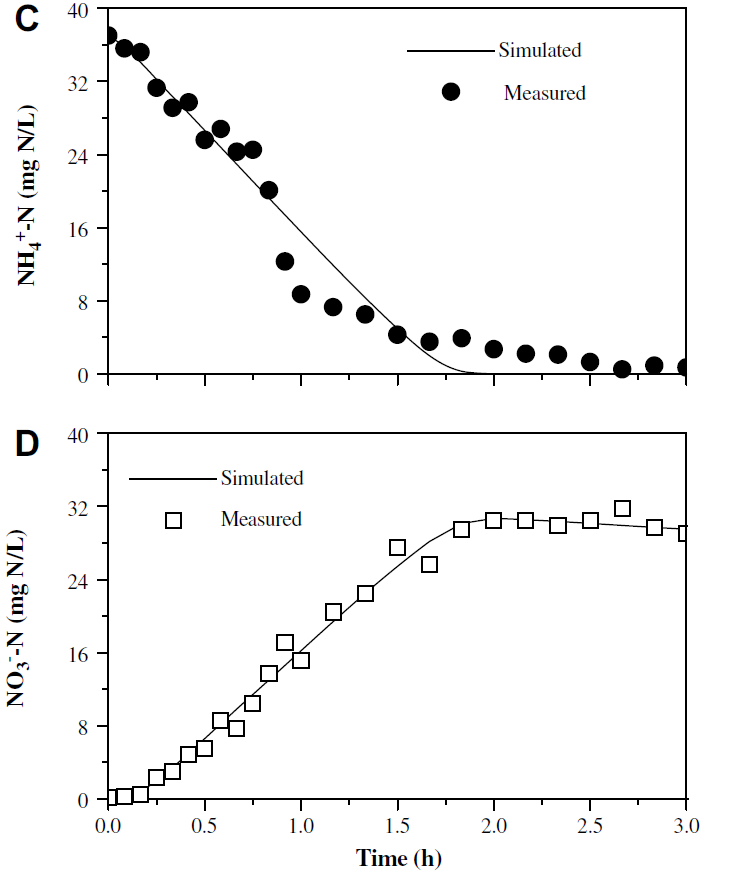


**Figure S7:** Figure 6 from Ni et al., 2009 showing the concentration profiles of various compounds during one SBR cycle. The corresponding DO was 2.00 mgO_2_ L^-1^.

**Table S7:** SND calculations for the SBR cycle shown in Figure S8. Plot Digitizer 2.6.8 was used to extract data from the figure Huwaldt, 2015.

| NH_4_-N start | NH_4_-N end | NO_3_-N start | NO_3_-N end | NH_4_-N removed | NO_3_-N accumulated | N denitrified | SND |
| --- | --- | --- | --- | --- | --- | --- | --- |
| mgN L^-1^ | mgN L^-1^ | mgN L^-1^ | mgN L^-1^ | mgN L^-1^ | mgN L^-1^ | mgN L^-1^ | % |
| 37.16 | 0.77 | 0 | 29.07 | 36.39 | 29.07 | 7.32 | 20.1 |


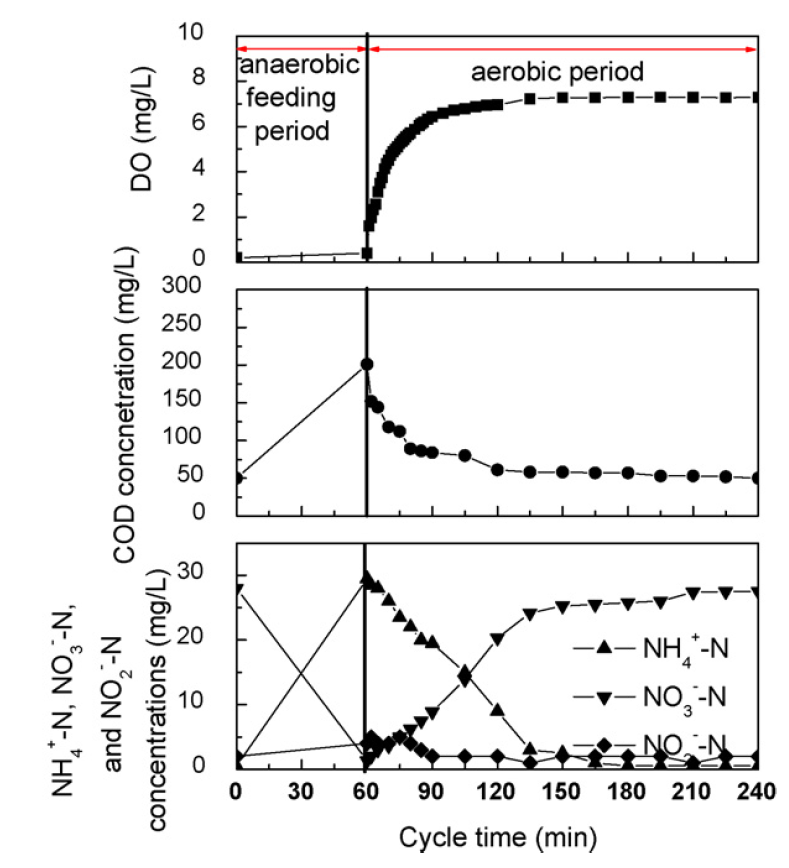


**Figure S8:** Figure 5 from Liu et al., 2010 showing the concentration profiles of various compounds during one SBR cycle. The corresponding DO was 7.3 mgO_2_ L^-1^.

**Table S8:** SND calculations for the SBR cycle shown in Figure S9. Plot Digitizer 2.6.8 was used to extract data from the figure Huwaldt, 2015.

| NH_4_-N start | NH_4_-N end | NO_3_-N start | NO_3_-N end | NH_4_-N removed | NO_3_-N accumulated | N denitrified | SND |
| --- | --- | --- | --- | --- | --- | --- | --- |
| mgN L^-1^ | mgN L^-1^ | mgN L^-1^ | mgN L^-1^ | mgN L^-1^ | mgN L^-1^ | mgN L^-1^ | % |
| 29.4 | 0.46 | 1.08 | 27.5 | 28.94 | 26.42 | 2.52 | 8.7 |


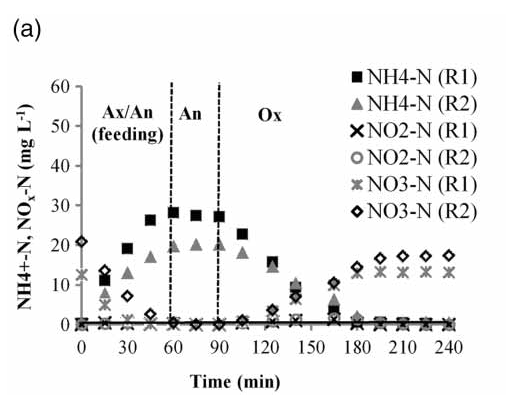


**Figure S9:** Figure 2a from Lashkarizadeh et al., 2015 showing the concentration profiles of various compounds during one SBR cycle. The corresponding DO was 9.1 mgO_2_ L^-1^.

**Table S9:** SND calculations for the SBR cycle shown in Figure S10. Plot Digitizer 2.6.8 was used to extract data from the figure Huwaldt, 2015.

| NH_4_-N start | NH_4_-N end | NO_3_-N start | NO_3_-N end | NH_4_-N removed | NO_3_-N accumulated | N denitrified | SND |
| --- | --- | --- | --- | --- | --- | --- | --- |
| mgN L^-1^ | mgN L^-1^ | mgN L^-1^ | mgN L^-1^ | mgN L^-1^ | mgN L^-1^ | mgN L^-1^ | % |
| 19.26 | 0 | 0 | 17.54 | 19.26 | 17.54 | 1.72 | 8.9 |

**Table S10:** SND calculations from N removal data provided in Rollemberg et al., 2019. Reactor 1, 2 and 3 were fed by acetate, glucose and ethanol as sole carbon source, respectively. Experimental phases 1, 2 and 3 were comprised of settling times 20, 10 and 5 min, respectively. The mean DO during aerobic conditions was estimated at 3.5 mgO_2_ L^-1^.

| Reactor | Exp.  Phase | NH_4_-N influent | NH_4_-N effluent | NO_2_-N effluent | NO_3_-N effluent | NO_x_-N start | NH_4_-N removed | NO_x_-N denitrified | SND | TN | Nitrifi-  cation |
| --- | --- | --- | --- | --- | --- | --- | --- | --- | --- | --- | --- |
|  |  | mgN L^-1^ | mgN L^-1^ | mgN L^-1^ | mgN L^-1^ | mgN L^-1^ | mgN L^-1^ | mgN L^-1^ | % | % | % |
| 1 | 1 | 108 | 22 | 1 | 0 | 1 | 86 | 85 | 99 | 79 | 80 |
|  | 2 | 115 | 46 | 9 | 0 | 9 | 69 | 60 | 87 | 52 | 60 |
|  | 3 | 108 | 34 | 13 | 2 | 15 | 74 | 59 | 80 | 55 | 69 |
| 2 | 1 | 104 | 26 | 3 | 1 | 4 | 78 | 74 | 95 | 71 | 75 |
|  | 2 | 115 | 37 | 7 | 1 | 8 | 78 | 70 | 90 | 61 | 68 |
|  | 3 | 112 | 49 | 6 | 6 | 12 | 63 | 51 | 81 | 46 | 56 |
| 3 | 1 | 112 | 49 | 12 | 6 | 18 | 63 | 45 | 71 | 40 | 56 |
|  | 2 | 110 | 9 | 10 | 4 | 14 | 101 | 87 | 86 | 79 | 92 |
|  | 3 | 108 | 34 | 31 | 8 | 39 | 74 | 35 | 47 | 32 | 69 |

# S2 AGS model description

The AGS model used in this study consists of three main components: (1) the biofilm model, (2) the biokinetic model and 3) the reactor model.

## Sumo Biofilm Model

*The SUMO biofilm model* is based on the 1-dimensional Wanner-Reichert mixed-culture biofilm model (Wanner and Reichert, 1996). The main difference between the Wanner-Reichert and the SUMO biofilm model is that the Wanner-Reichert biofilm model predicts (calculates) the biofilm thickness, while in the SUMO biofilm model the biofilm thickness (or layer thickness) is an input (constant). Also, the total granule volume is considered constant in the model (default 300 m^3^ of 5000 m^3^ total reactor volume). The SUMO biofilm model sub-divides the compartments into bulk (compartment #0) and biofilm (composed of granule layers #1 to #10). Bulk and biofilm are “separated” by the boundary layer (default thickness 50 µm). The mass-transfer mechanism affecting soluble / colloidal compounds (S_i_ and C_i_) is diffusion only, while the particulate compounds (X_i_) are affected by the mass-transfer mechanisms displacement, attachment and internal transfer. The specific differential equations and concepts of the mass-transfer mechanisms are detailed in Wanner and Reichert, 1996. Mass-transfer of S_i_ and C_i_ by diffusion is based on Fick’s first law of diffusion and occurs between all compartments.
Mass-transfer of X_i_ is based on displacement, attachment and internal transfer. Displacement transfers X_i_ by an advective flux between all compartments. The driving force for mass-transfer by displacement is the difference between the maximum TSS concentration allowed within any compartment (102 kgTSS m^-3^_compartment_) and the actual TSS concentration within the compartment. The maximum TSS (or biofilm density) is calculated from dry matter content of biofilm (0.1 kg kg^-1^) multiplied by the biofilm density (1020 kg m^-3^_compartment_), which corresponds to 102 kgTSS m^-3^_compartment_ (concentration in the biofilm per unit volume biofilm). The values used for calculations are default values provided within the AGS model of SUMO (Table S12), and reflect typically applied values in biofilm / AGS modelling (30 – 450 kgTSS m^-3^_compartment_, Lee and Park, 2007; Elenter et al., 2007; Rittmann et al., 2018; Vázquez-Padín et al., 2010). Those values are within the range of densities experimentally measured for granules fed by synthetic or municipal WW (30-150 kgTSS m^-3^, Nor-Anuar et al., 2012). Attachment transfers X_i_ from the bulk to the granule surface compartments only. Internal-transfer is a flux of X_i_ that only occurs within the biofilm compartments (between granule layers 1 to 10). It is based on Fick’s first law of diffusion, but uses an internal solids transfer rate (effective diffusive flux) similar for all X_i_ (default 10^-14^ cm^2^ s^-1^). Table S11 and S12 provide detailed information on the calculation of the different mass-transfer mechanisms implemented, and all parameters used in the SUMO biofilm model, respectively.

## Biokinetic model

*The SUMO1 biokinetic model* (Varga et al., 2018) was used in our study and includes the following key processes:

(1) Growth and decay of the following microbial populations: ordinary heterotrophic organisms (OHO), phosphorus accumulating organisms (PAO), glycogen accumulating organisms (GAO), nitrifying organisms (NITO), among other organisms groups that are strictly anaerobic and thus inhibited under partially aerobic conditions as in this study (methylotrophic organisms (MEOLO)), acidoclastic methanogens (AMETO), hydrogenotrophic methanogens (HMETO)).
(2) Nitrification and denitrification are modelled as 1-step processes. Nitrification is performed by NITO. Denitrification can be performed by OHO, GAO and PAO. Denitrification by OHO is performed by using VFA or S_B_ as electron-donors. GAO can perform denitrification by using internally stored GLY (glycogen), and PAO denitrify using PHA.
(3) Fermentation of S_B_ to VFA can be performed by OHO and PAO.
(4) Hydrolysis of particulate organic substrate (X_B_) is redox-sensitive, *i.e.*, decreased under anoxic or anaerobic conditions (similar to ASM2d, Henze et al., 2000).
Table S13 provides the full biokinetic matrix and parameters of the biokinetic model SUMO1.

## Reactor model

*The reactor model* used in this study is a sequencing batch reactor (SBR). The SBR sequence (total 6 h) consists of anaerobic filling (0.1 h), anaerobic mixing (1.4 h), aeration (4.05 h), settling and sludge withdrawal (0.45 h combined). The volume exchange ratio (VER) is 30%. All process steps are modelled in fully-mixed conditions (no plug-flow, no settling). Effluent TSS is a user input (default 20 mgTSS L^-1^). SRT is calculated according to Equation (1).
${SRT}_{target}= \frac{{TSS}_{r}\cdot V_{r}}{{TSS}_{eff}\cdot Q_{eff}+{TSS}_{bulk}\cdot Q_{ex}}$ (1)
TSS_r_ is the TSS concentration in the reactor (gTSS L^-1^), V_r_ is the reactor volume (L), TSS_eff_ is the TSS concentration in the effluent (gTSS L^-1^), Q_eff_ is the effluent flow rate (L d^-1^), TSS_bulk_ is the TSS concentration in the bulk compartment (gTSS L^-1^) and Q_ex_ is the excess sludge flow rate (L d^-1^). Q_ex_ was automatically calculated based on SRT_target_ = 20 d. Excess sludge is only withdrawn from the bulk compartment and no granules are wasted.

*Specific model adaptations* included the selection of granule diameters 0.5 and 2.0 mm resembling young and mature granules, respectively. Also, the granule compartment was discretised by 10 biofilm layers. In the case of 0.5 mm diameter granules, all layers were of equal thickness (25 µm). In the case of 2.0 mm diameter granules, the 4 outermost (surface) biofilm layers were set to 25 µm in thickness, while the remaining 6 inner biofilm layers were set to 150 µm in thickness. Decreasing the thickness of the 4 outermost (surface) biofilm layers of the 2.0 mm diameter granules was done to increase the resolution in those layers, and to equate both granule sizes in terms of surface biofilm layers thickness.

*The AGS model* implemented in SUMO combines mass balances of soluble (S_i_), colloidal (C_i_) and particulate (X_i_) compounds induced by the mass-transfer processes of the biofilm model (diffusion, displacement, attachment and internal transfer), biokinetics (biological reaction rates) and reactor model (influent, effluent, excess sludge removal).

**Table S11**: Mass transfer processes diffusion, displacement, attachment and internal transfer in the SUMO® biofilm model. Default values were used where applicable.

| Process | Calculations |
| --- | --- |
| Diffusion  (S_i_, C_i_) | Diffusion (bulk to granule) = -D_F,SCi_*A_GS_*(SC_i,n_-SC_i,n+1_)/z_BL_ [g d^-1^] Diffusion (inside granule) = -D_F,SCi_*A_LS,n_*(SC_i,n_-SC_i,n+1_)/z_L,n_ [g d^-1^]  D_F,SCi_ = D_SCi_*f_D_*8.64 effective diffusion coefficient of dissolved or colloidal compound SC_i_ [m^2^ s^-1^] D_Si_ = diffusion coefficient of dissolved or colloidal compound SC_i_ [m^2^ s^-1^] f_D_ = empirical reduction factor of diffusion rate in biofilm [default 0.5] A_GS_, A_LS,n_ = granule surface area, granule layer n surface area [m^2^] SC_i,n_ = concentration of dissolved or colloidal compound i in layer n [g m^-3^] z_BL_, z_L,n_ = boundary layer thickness [default 5*10^-5^ m], granule layer n thickness [m] |
| Displacement (X_i_) | Displacement (bulk to granule) =  If X_TSS,b_ > X_TSS,GS_ 🡪 -r_dpm,b_*f_XTSS,Xi,b_ [g d^-1^];  else 🡪 -r_dpm,GS_*f_XTSS,Xi,GS_*A_GS_*(X_TSS,n_-X_TSS,n+1_)/z_BL_ [g d^-1^]; Displacement (inside granule) =  If X_TSS,n_ > X_TSS,n+1_ 🡪 -r_dpm,n_*f_XTSS,Xi,n_*A_LS,n+1_*(X_TSS,n_-X_TSS,n+1_)/z_L,n_ [g d^-1^];  else 🡪 -r_dpm,n+1_*f_XTSS,Xi,n+1_*A_LS,n+1_*(X_TSS,n_-X_TSS,n+1_)/z_L,n_ [g d^-1^];  X_TSS,b_, X_TSS,GS_, X_TSS,n_ = TSS concentration in bulk, granule surface, granule layer n [g m^-3^] f_XTSS,Xi,b_, f_XTSS,Xi,GS_, f_XTSS,Xi,n_ = fraction of concentration of particulate compound X_i_ in concentration of TSS in bulk, granule surface, granule layer n [-] r_dpm,b,_ r_dpm,GS_ = displacement rate bulk, granule surface [g d^-1^] r_dpm,n_ = r_dpm,max_*1/(1+exp(-s_I_*(X_TSS,n_-X_TSS,max_))) displacement rate in granule layer n [g d^-1^] r_dpm,max_ = displacement rate of solids between biofilm layers [default 10^-6^ m d^-1^] s_I_= Slope of switching function around X_TSS,max_ [default 0.004 m^3^ g^-1^] X_TSS,max_= X_TSS,F_*ρ_F_ maximum TSS concentration in biofilm [default 102 kg m^-3^] X_TSS,F_ = dry matter content of biofilm [default 0.1 kg kg^-1^] ρ_F_ = biofilm density [default 1020 kg m^-3^] A_GS_, A_LS,n+1_ = granule surface layer area, granule layer n+1 surface area [m^2^] z_BL_, z_L,n_ = boundary layer thickness [default 5*10^-5^ m], granule layer n thickness [m] |
| Attachment (X_i_) | Attachment = -r_att_*A_GS_*X_i,b_  [g d^-1^]  r_att_= attachment rate [default 5*10^-2^ m d^-1^] A_GS_ = granule surface area [m^2^] X_i,b_ = concentration of particulate compound i in bulk [g m^-3^] |
| Internal transfer (X_i_) | Transfer = -D_F,X_*A_LS,n+1_*(X_i,n_-X_i,n+1_)/z_L,n_ [g d^-1^]  D_F,X_= D_X_*8.64 D_X_ = internal solids transfer rate in biofilm [default 10^-14^ cm^2^ s^-1^] A_LS,n+1_= granule layer n+1 surface area [m^2^] X_i,n_, X_i,n+1_ = concentration of particulate compound i in granule layer n, n+1 [g m^-3^] z_L,n_ = granule layer n thickness [m] |

**Table S12:** List of parameters as part of the SUMO biofilm model used in this study.

| Parameter name | Abbreviation | Value | Unit |
| --- | --- | --- | --- |
| Empirical reduction factor of diffusion rate in biofilm | f_D_ | 0.5 | - |
| Internal enthalpy transfer rate in biofilm | D_H_ | 0 | cm^2^.s^-1^ |
| Internal solids transfer rate in biofilm | D_X_ | 1.0E-14 | cm^2^.s^-1^ |
| Attachment rate of solids to biofilm | r_att_ | 0.05 | m.d^-1^ |
| Detachment rate of solids from biofilm | r_det_ | 0 | m.d^-1^ |
| Displacement rate of solids between biofilm layers | r_dpm,max_ | 1.0E-06 | m.d^-1^ |
| Net volume occupied by the granules | V_gran_ | 300 | m^3^ |
| Boundary layer thickness | z_BL_ | 5.0E-05 | m |
| Dry matter content of biofilm | X_TSS,F_ | 0.1 | kg kg^-1^ |
| Biofilm density | ρ_F_ | 1020 | kg m^-3^ |
| Slope of switching function around XTSS_max_ | s_l_ | 4.0E-03 | m^3^ g^-1^ |

**Table S13:** Kinetic (Gujer) matrix, model parameters and calculated variables of the mathematical model SUMO1 used in this study (Varga et al., 2018). The tables are in “multimedia component 1”.

**Table S14**: Influent fractionation of municipal wastewater (MWW) and 100% VFA synthetic wastewater (VFA) used for the SUMO® simulations.

| Influent fractions | MWW | VFA |  |
| --- | --- | --- | --- |
| Filtered COD fraction (incl. colloids, VFA) | 40.5 | 100 | % |
| Filtered flocculated COD fraction (incl. VFA) | 20.2 | 100 | % |
| VFA fraction of filtered COD | 11.8 | 100 | % |
| Unbiodegradable filtered COD fraction | 11.8 | 0 | % |
| Influent particulate inert COD fraction | 14.0 | 0 | % |
| Influent heterotrophic fraction of COD | 5.0 | 0 | % |
| Influent endogenous products fraction of OHOs | 20.0 | 0 | % |
| Unbiodegradable fraction of influent colloids | 20.0 | 0 | % |
| Ammonia fraction of TKN | 69.8 | 100 | % |
| Phosphate fraction of TP | 58.1 | 100 | % |
| N fraction of filtered biodegradable COD | 4.0 | 0 | % |
| N fraction of unbiodegradable COD | 1.0 | 0 | % |
| P fraction of filtered biodegradable COD | 1.0 | 0 | % |
| P fraction of unbiodegradable COD | 0.1 | 0 | % |

**Table S15:** Processes linked to electron-donor utilisation in anaerobic, anoxic and aerobic redox conditions by OHO, PAO and GAO, respectively. More details can be found in the full biokinetic matrix Supplementary Information Table S13.

| Redox condition | OHO | PAO | GAO |
| --- | --- | --- | --- |
| Anaerobic | - Growth on S_B_ at high VFA conc. - Growth on S_B_ at low VFA conc. | - PHA storage from VFA - Growth via S_B_ at high or low VFA conc. | - GLY storage from VFA - Maintenance on GLY |
| Anoxic | - Growth on S_B_ - Growth on VFA | - Growth on PHA - Growth on PHA (PO_4_^3-^ limited) - Maintenance on PHA | - Growth on GLY - Maintenance on GLY |
| Aerobic | - Growth on S_B_ - Growth on VFA | - Growth on PHA - Growth on PHA (PO_4_^3-^ limited) - Maintenance on PHA | - Growth on GLY - Maintenance on GLY |

**References:**

ELENTER, D., MILFERSTEDT, K., ZHANG, W., HAUSNER, M. & MORGENROTH, E. 2007. Influence of detachment on substrate removal and microbial ecology in a heterotrophic/autotrophic biofilm. *Water Research,* 41**,** 4657-4671.

HE, Q. L., CHEN, L., ZHANG, S. J., CHEN, R. F. & WANG, H. Y. 2019. Hydrodynamic shear force shaped the microbial community and function in the aerobic granular sequencing batch reactors for low carbon to nitrogen (C/N) municipal wastewater treatment. *Bioresource Technology,* 271**,** 48-58.

HENZE, M., GUJER, W., MINO, T. & VAN LOOSDRECHT, M. C. M. 2000. *Activated Sludge Models ASM1, ASM2, ASM2d and ASM3*, IWA Publishing.

HUWALDT, J. A. 2015. Plot Digitizer. 2.6.8 ed.: <http://plotdigitizer.sourceforge.net/>.

LASHKARIZADEH, M., YUAN, Q. & OLESZKIEWICZ, J. A. 2015. Influence of carbon source on nutrient removal performance and physical-chemical characteristics of aerobic granular sludge. *Environmental Technology (United Kingdom),* 36**,** 2161-2167.

LEE, M. W. & PARK, J. M. 2007. One-dimensional mixed-culture biofilm model considering different space occupancies of particulate components. *Water research,* 41**,** 4317-4328.

LIU, Y.-Q., KONG, Y., TAY, J.-H. & ZHU, J. 2011. Enhancement of start-up of pilot-scale granular SBR fed with real wastewater. *Separation and Purification Technology,* 82**,** 190-196.

LIU, Y.-Q., MOY, B., KONG, Y.-H. & TAY, J.-H. 2010. Formation, physical characteristics and microbial community structure of aerobic granules in a pilot-scale sequencing batch reactor for real wastewater treatment. *Enzyme and Microbial Technology,* 46**,** 520-525.

NI, B.-J., XIE, W.-M., LIU, S.-G., YU, H.-Q., WANG, Y.-Z., WANG, G. & DAI, X.-L. 2009. Granulation of activated sludge in a pilot-scale sequencing batch reactor for the treatment of low-strength municipal wastewater. *Water Research,* 43**,** 751-761.

NOR-ANUAR, A., UJANG, Z., VAN LOOSDRECHT, M. C. M., DE KREUK, M. K. & OLSSON, G. 2012. Strength characteristics of aerobic granular sludge. *Water Science and Technology,* 65**,** 309-316.

PRONK, M., DE KREUK, M. K., DE BRUIN, B., KAMMINGA, P., KLEEREBEZEM, R. & VAN LOOSDRECHT, M. C. M. 2015. Full scale performance of the aerobic granular sludge process for sewage treatment. *Water Research,* 84**,** 207-217.

RITTMANN, B. E., BOLTZ, J. P., BROCKMANN, D., DAIGGER, G. T., MORGENROTH, E., SØRENSEN, K. H., TAKÁCS, I., VAN LOOSDRECHT, M. & VANROLLEGHEM, P. A. 2018. A framework for good biofilm reactor modeling practice (GBRMP). *Water Science and Technology,* 77**,** 1149-1164.

ROLLEMBERG, S. L. D. S., DE OLIVEIRA, L. Q., BARROS, A. R. M., MELO, V. M. M., FIRMINO, P. I. M. & DOS SANTOS, A. B. 2019. Effects of carbon source on the formation, stability, bioactivity and biodiversity of the aerobic granule sludge. *Bioresource Technology,* 278**,** 195-204.

ŚWIĄTCZAK, P. & CYDZIK-KWIATKOWSKA, A. 2018. Performance and microbial characteristics of biomass in a full-scale aerobic granular sludge wastewater treatment plant. *Environmental Science and Pollution Research,* 25**,** 1655-1669.

VARGA, E., HAUDUC, H., BARNARD, J., DUNLAP, P., JIMENEZ, J., MENNITI, A., SCHAUER, P., LOPEZ VAZQUEZ, C. M., GU, A. Z., SPERANDIO, M. & TAKÁCS, I. 2018. Recent advances in bio-P modelling – a new approach verified by full-scale observations. *Water Science and Technology,* 78**,** 2119-2130.

VÁZQUEZ-PADÍN, J. R., MOSQUERA-CORRAL, A., CAMPOS, J. L., MÉNDEZ, R., CARRERA, J. & PÉREZ, J. 2010. Modelling aerobic granular SBR at variable COD/N ratios including accurate description of total solids concentration. *Biochemical engineering journal,* 49**,** 173-184.

WAGNER, J., GUIMARÃES, L. B., AKABOCI, T. R. V. & COSTA, R. H. R. 2015. Aerobic granular sludge technology and nitrogen removal for domestic wastewater treatment. *Water Science and Technology,* 71**,** 1040-1046.

WANNER, O. & REICHERT, P. 1996. Mathematical modeling of mixed-culture biofilms. *Biotechnology and Bioengineering,* 49**,** 172-184.
